# Supplementary material for: Leaky or polarised immunity: Non-Markovian modelling highlights the impact of immune memory assumptions
Source: PLoS Comput Biol. 2025 Aug 19;21(8):e1013399. doi: 10.1371/journal.pcbi.1013399 (PMC12407549; doi:10.1371/journal.pcbi.1013399)
Supplement: S1 Text — (PDF) [file pcbi.1013399.s001.pdf]

Supplementary material

Leaky or polarised immunity: non-Markovian modelling highlights the impact of immune memory assumptions

Bastien Reyné[✉]

Tsukushi Kamiya

Ramsès Djidjou-Demasse[=]

Samuel Alizon[=]

Mircea T. Sofonea[✉]

= equal contribution

✉ corresponding authors : reyne.bastien@gmail.com
mircea.sofonea@umontpellier.fr

A Supplementary methods and results

A.1 Induction up to time $t = 2$

We assume a decreasing function ξ that represents the immune efficacy in the two models of partial immunity (i.e., leaky and polarised immunity). The function ξ depends on the age of immunity. We follow a cohort of individuals that acquired their immunity on the same date τ, that will be exposed to the same (and constant) force of infection, Λ. We assume that the initial date is time $\tau = 0$, and we further assume we have a normalised quantity of individuals in both models of immunity, $q_\ell(0) = q_p(0) = 1$.

In the leaky model, $\xi(\tau)$ represents the decrease in susceptibility after acquiring the immunity τ days ago. At time $\tau = 1$, the proportion of uninfected individuals is given by

$$q_\ell(1) = q_\ell(0) - q_\ell(0) \cdot [1 - \xi(0)] \cdot \Lambda = q_\ell(0)[1 - \Lambda \cdot [1 - \xi(0)]] = 1 - \Lambda \cdot [1 - \xi(0)]. \quad \text{(S1)}$$

In the polarised model, $\xi(\tau)$ represents the fraction of completely immune individuals. Consequently, there are fully susceptible individuals, which means we may write at each time step $q_p(\tau) = \xi(\tau) + s(\tau)$ where s represents fully susceptible individuals. At time $\tau = 1$, we have (thanks to Equation (2))

$$q_p(1) = q_p(0)[1 - \Lambda] + \xi(0)\Lambda = 1 - \Lambda[1 - \xi(0)], \quad \text{(S2)}$$

which verifies $q_\ell(1) = q_p(1)$.

Using the induction Equation (1), we obtain for the leaky formalism at time $\tau = 2$,

$$q_\ell(2) = q_\ell(1)[1 - \Lambda] + q_\ell(1) \cdot \Lambda \cdot \xi(1). \quad \text{(S3)}$$

For the polarised formalism, it is given by

$$q_p(2) = q_p(1)[1 - \Lambda] + \Lambda\xi(1). \quad \text{(S4)}$$

From equations S3-S4 and the fact that $q_\ell \leq 1$ (it is a proportion), we obtain that $q_\ell(2) < q_p(2)$ if $\Lambda > 0$, and $q_\ell(2) = q_p(2)$ if $\Lambda = 0$, which guarantees our inequality

$$q_\ell(2) \leq q_p(2). \quad \text{(S5)}$$

A.2 Immunity efficacy functions

We consider four different shapes for the function $f(a)$ representing the imperfect immunity function, which is a decrease in susceptibility for recovered individuals in the leaky model and the proportion of individuals still immune in the polarised paradigm. In every case, we assume (partial) immunity to last 600 days, which is in line with the order of magnitude observed for SARS-CoV-2 (*cf.* Stein *et al.* [2023] and Appendix E). The main difference lies in the shape of the function, as represented in Figure 1. The functions used are

$$f_1(a) = \max_{a \in \mathbb{R}_+} \left\{ 0 \quad ; \quad \frac{g_1(a) - g_1(600)}{g_1(0) - g_1(600)} \right\} \qquad \text{with} \quad g_1(a) = \frac{1}{1 + \exp\left(\frac{a-300}{100}\right)},$$

$$f_2(a) = \max_{a \in \mathbb{R}_+} \left\{ 0 \quad ; \quad 1 - \frac{a}{600} \right\},$$

$$f_3(a) = \max_{a \in \mathbb{R}_+} \left\{ 0 \quad ; \quad \frac{g_3(a) - g_3(600)}{g_3(0) - g_3(600)} \right\} \qquad \text{with} \quad g_3(a) = \frac{a^3}{600},$$

$$f_4(a) = \max_{a \in \mathbb{R}_+} \left\{ 0 \quad ; \quad \frac{g_4(a) - g_4(600)}{g_4(0) - g_4(600)} \right\} \qquad \text{with} \quad g_4(a) = 1 - \frac{a^{0.5}}{600},$$

$$f_5(a) = \begin{cases} 1 - \frac{a}{400} & \text{if} \quad a < 200, \\ 0.5 & \text{if} \quad 200 < a < 400, \\ 0.5 - \frac{a}{400} & \text{if} \quad 400 < a < 600, \\ 0 & \text{if} \quad a > 600. \end{cases}$$

Note the precise definition of these functions (and the fact they are scaled) is not important *per se*, as they were chosen to show different shapes of functions. We selected a constant force of infection, $\Lambda = 0.005$, which is an order of magnitude coherent with the SARS-CoV-2 pandemic. The results are presented in the Figure 1.

A.3 Fitted force of infection

We display on Figure A the fitted force of infection for the two models of partial immunity. Importantly, we did not include any Omicron sub-variants while they kept appearing during the time in our simulations. Therefore, the force of infection values past March 2022 should be taken with caution as the models need to fit a high force of infection to counterbalance the absence of immune escape properties.

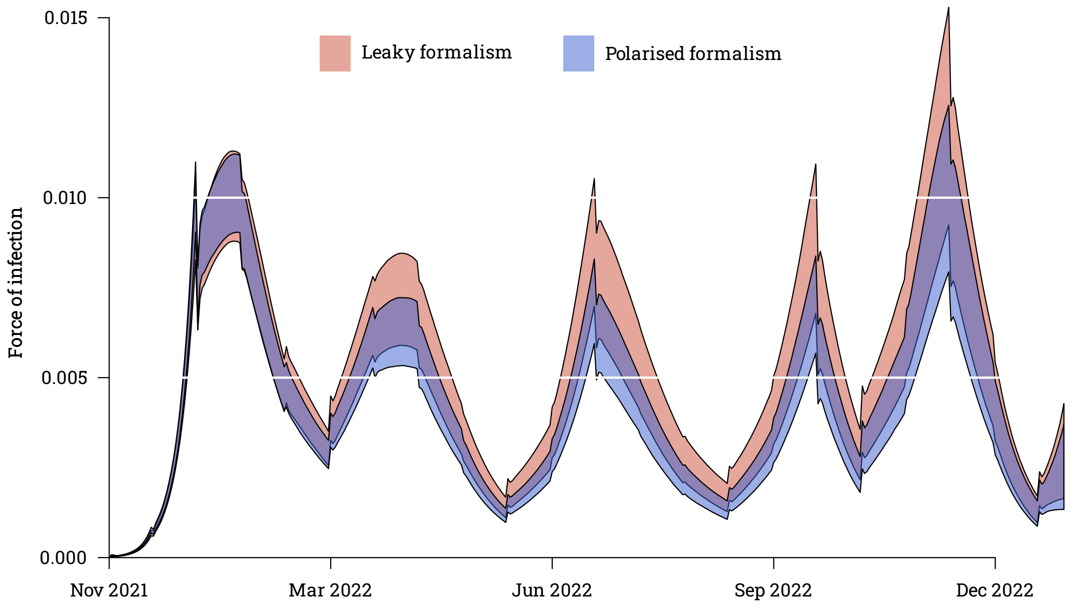

FIGURE A: Fitted force of infection in both models.

B Non-Markovian basics

B.1 A brief and non-exhaustive history of non-Markovian models in epidemiology

In infectious disease modelling, non-Markovian representations of biological processes are almost as old as the discipline itself. As early as in 1927, KERMACK and MCKENDRICK [1927] discussed the natural stage of infection over time in their seminal paper. The elementary *susceptible – infected – recovered* (*SIR*) model for which this study is famous was introduced only as a special case, assuming exponentially-distributed residence waiting times. Five years later, the same Kermack & McKendrick published a follow-up paper where they introduced a model based on partial differential equations (PDEs) [KERMACK and MCKENDRICK 1932]. This formalism has the advantage of recording explicitly the time spent in each compartment by adding a second time-variable to each compartment. The ability to explicitly account for a non-exponentially distributed waiting time in a state is what we call non-Markovian.

However, it has an extra cost in terms of complexity as it requires each compartment to have a boundary condition (accounting for the entry in the compartment) and a differential equation (accounting for the time-dependent transition rate). With notations consistent with

contemporary literature (and some little simplifications), we could write their model with the following equations. In particular, all three compartments (susceptible, infected, recovered) use a non-Markovian structure. Individuals enter the susceptible compartment at time a following the boundary condition

$$S(t, a = 0) = \mu, \tag{S6}$$

where μ represents both migration and demography. As recovered individuals are reinfected, they enter the infected compartment (at time $\tau = 0$) without going through the susceptible compartment. It results, the newly infected individuals accounts for both susceptible and recovered individuals:

$$I(t, \tau = 0) = \lambda(t) \left[\int_0^\infty S(t, a) \mathrm{d}a + \int_0^\infty [1 - \xi(s)] R(t, s) \mathrm{d}s \right], \tag{S7}$$

where $\lambda(t) = \int_0^\infty \omega(\tau) I(t, \tau) \mathrm{d}\tau$ is the force of infection ($\omega(\cdot)$ is the generation time density of probability), $R(t, s)$ is the recovered since s units of time and $\xi(s)$ the decrease in susceptibility for individuals recovered since s units of time. Following the same logic, individuals enter the recovered infection (at $s = 0$)

$$R(t, s = 0) = \int_0^\infty \gamma(\tau) I(t, \tau) \mathrm{d}\tau, \tag{S8}$$

where $\gamma(\tau)$ is the recovery rate for infected individuals after τ units of time. These three equations are coupled with the system

$$\begin{cases} \left(\dfrac{\partial S(t, a)}{\partial t} + \dfrac{\partial S(t, a)}{\partial a} \right) = -\lambda(t)\, S(t, a), \\ \left(\dfrac{\partial I(t, \tau)}{\partial t} + \dfrac{\partial I(t, \tau)}{\partial \tau} \right) = -\left[\gamma(\tau) + \alpha(\tau) \right] I(t, \tau), \\ \left(\dfrac{\partial R(t, s)}{\partial t} + \dfrac{\partial R(t, s)}{\partial s} \right) = -\lambda(t)\, [1 - \xi(s)]\, R(t, s), \end{cases} \tag{S9}$$

where α is the disease-induced mortality rate. The whole set of Equations S6–S9 is the non-Markovian counterpart of the elementary *SIR* model. For each compartment where we record the residence time (*e.g.* the age of infection) with a second time structure (*e.g.* τ), we need to add a boundary equation that accounts for the entry in the compartment (*e.g.* at time $\tau = 0$). That allows us to make parameters such as the recovery rate (γ) time-dependent.

The gain in precision has a cost from an analytical (or numerical) analysis, which may explain why this formalism did not gain as much popularity as the simpler *SIR* model.

Nonetheless, it remains one of the first models to account explicitly for time-dependent biological processes.

Non-Markovian modelling has manifested itself through a wide variety of approaches over time with some that are well known and others more obscure. Examples include discrete-time models with a fixed delay [Wilson and Burke 1942] or models based on delayed differential equations [Yorke 2006] that eventually are not commonly used today. However, these concepts have evolved—an exposed compartment in a *SEIR* model, for instance, effectively introduces a delay that reflects intra-host pathogen replication prior to inter-host transmission, aligning the model more closely with the pathogen's life history. In addition,a method that remains popular is the so-called chain trick [Blythe and Anderson 1988] that relies on the hypoexponential distribution as waiting times in different states [Hethcote, Stech, and Van Den Driessche 1981; Lloyd 2001], allowing to approximate some empirical distributions without departing from the ordinary differential equations paradigm.

B.2 A brief overview of non-Markovian formalisms

We may encounter several ways to implement non-Markovian properties in transmission models based on different mathematical formalisms. Here, we introduce the use of non-Markovian models in epidemiology without being exhaustive. *Stricto sensu*, a model is non-Markovian when at least one process is characterised by a time-dependent (relative to the time spent in the compartment) parameter. Indeed, a constant departure rate from a compartment (in an ODEs-based model) would imply Markovian, *i.e.*, a residence time that follows an exponential distribution. The classical force of infection $\lambda(t) := \beta\, I(t)$ does not depend on the time spent in $S(t)$ or $I(t)$, only on the state of the system at a given time (autonomy). The *SIR* model with a constant recovery rate is therefore Markovian. The same goes for the *SIRS* model if the loss of immunity is characterised by a constant rate.

Now, take the *SEIR* model. It was designed to implement a delay between infection and contagiousness by incorporating a latency period with the *exposed* compartment. From a technical point of view, it does not depart from the *SIR* model, but with an extra parameter that is also a constant. However, *SEIR* model is non-Markovian, as the exposed and infected states represent a unique epidemiological state (an infection, from its exposure to its recovery). The

added compartment E changes both the generation time and the residence time distribution [Svensson 2007]. The addition of the E compartment could be seen as the first chain trick and following this, every model that would use a chain trick (as described by Blythe and Anderson [1988]) under the form $S \rightarrow I_1 \rightarrow \cdots \rightarrow I_k \rightarrow R$ could also be characterised non-Markovian. (It is also true if the chain trick is implemented on any other state rather than infected.)

Besides the chain trick in ODEs-based models (Hethcote, Stech, and Van Den Driessche [1981] is a great example), there are alternative formalisms that present non-Markovian properties. We may mention the PDE-based models that were designed in the first place to incorporate heterogeneous waiting time distributions or time-dependent transmission rate (*cf.* Appendix B). In addition, we could also mention non-Markovian discrete-time non-Markovian models where at each time step, individuals move to the following time state with their rate of departure (such as in this study, *cf.* Models and method, or in Diekmann *et al.* [2021] and Sofonea, Reyné, *et al.* [2021]). Finally, there are individual (also called agent)-based models where the departure rates from compartments are drawn within a specified distribution which adds some heterogeneity to the waiting time distributions (*e.g.* Elie, Selinger, and Alizon [2022]). To our knowledge, these four aforementioned paradigms represent the most predominant and common ways to implement non-Markovian models in epidemiology.

In this study, we will refer to non-Markovian models when at least one distribution related to an order-one kinetic (such as contagiousness, recovery or death but not transmission or reproduction *e.g.*) departs explicitly from the exponential distribution. Some formalisms would fall directly in this category such as PDEs, Equations S6–S9 make clear that the different biological processes are time-dependant and follow a particular distribution (*e.g.* $\gamma(\cdot)$, $\xi(\cdot)$ and $\omega(\cdot)$), or discrete-time non-Markovian models.

B.3 Our heuristic for selecting non-Markovian formalisms

As seen in Section B.2, there are various implementations of non-Markovian processes in epidemiology. Every choice may be genuinely adopted, but we believe there are some cases where some formalisms are more justified than others. The choice of a paradigm over another might

depend on many factors such as ease of implementation, ease of parametrisation, results interpretation, the possibility of analytical results or accounting for variance. For instance, models based on ODEs systems are convenient to implement numerically, are widely used among the modellers community, and are convenient to retrieve analytical results. However, when it comes to implementing a generation time following a given distribution (usually Gamma, Weibull or LogNormal), the user must first determine the right number of compartments and the associated parameters to reproduce empirically the targeted distribution. While it remains possible, it becomes more complicated when there are two competing processes happening on the same compartments (*e.g.,* implement leaky immunity would require accounting for both the age of immunity through chaining compartments and to have a specific decrease of susceptibility associated to each recovered compartment. Explicit distributions of the two competing phenomena are not straightforward.) Models based on PDEs provide advantages when it comes to implementing parametrically some biological processes, as such a task is straightforward. They can also be used to provide analytical results, but are more technical to implement as there is no (for now) direct implementation available (in R, at least), requiring the implementation of a manual Euler scheme. Discrete-time models are easier to implement than PDEs, but the analytical results are less evident as the continuous paradigm is discarded. However, it probably remains the best alternative in applied contexts when analytical results are not sought, and multiple non-Markovian processes intervene sufficiently to render the ODE option too cumbersome. Finally, agent-based models have the advantage of flexible implementation of the variance in the trajectories and stochasticity — albeit as the cost of a greater computational burden.

Ultimately, individual modellers may weigh the pros and cons of each approach. Our intention with this Appendix was to remind readers that ODE-based models may not be the best solution to all problems.

C Discrete-time approximations

The discrete-time *SIR* model (and its variations) is thought to be the discrete counterpart of the continuous *SIR* model first introduced by Kermack and McKendrick [1927]. However, the discretisation of the continuous time space induces some key assumptions.

Particularly, all the rates that appeared originally in the ODE system become probabilities in the discrete-time version. For instance, the recovery rate (that drives the leaving of the infected compartment) becomes the daily probability of recovery—assuming a daily discretisation of the time space.

On a related note, the force of infection that is the rate with which susceptible individuals become infected becomes the daily probability of infection *per capita*. As pointed out by some authors (*e.g.* Diekmann *et al.* [2021] and Keeling and Rohani [2008]), the original equation from the continuous *SIR*,

$$dS/dt = -\lambda(t)S(t) \tag{S10}$$

(where λ is the force of infection), is not equivalent to the discrete version term-to-term translation

$$S_{t+1} = S_t - \Lambda_t S_t \tag{S11}$$

(with Λ the discrete force of infection). Indeed, as the contact rate (β) becomes a daily probability *per capita*, the possibility of many encounters with infected individuals for a susceptible within the same time step may occur, meaning that a same individual could be infected twice in the same time span and need to be addressed in the discrete-time version. The true discrete-time version of Equation S10 should then be

$$S_{t+1} = S_t \exp(-\Lambda_t), \qquad \text{where } \Lambda_t := \int_t^{t+1} \lambda(s)ds \tag{S12}$$

as rightfully explained in details by Diekmann *et al.* [2021].

The careful reader will have noticed that we nevertheless used in the main text (and the numerical analyses) Equation S11 instead of Equation S12. Equation S11 remains a good approximation (first order Taylor development) with relatively low values of the force of infection [Diekmann *et al.* 2021], as in the SARS-CoV-2 case [Sofonea, Reyné, *et al.* 2021]. As we fit the contact rates on empirical data, the numerical inaccuracies that would theoretically arise (negative amount of susceptible individuals if the force of infection is too high) are unlikely. The used Equation S11 also provides a more convenient framework to fit contact rates as it does not involve exponentiation of integrals.

D Model equations

We implemented the leaky model using the following set of equations:

$$R^{\mathrm{D}}_{t+1,0} = 0, \tag{S13}$$

$$R^{\mathrm{D}}_{t+1,a+1} = [1 - \Lambda_t\,(1 - \xi^{\mathrm{D}}_a)]R^{\mathrm{D}}_{t,a}, \qquad \text{for } a \in [\![1, \dots, N_a - 1]\!], \tag{S14}$$

$$R^{\mathrm{D}}_{t+1,N_a} = R^{\mathrm{D}}_{t,N_a} + [1 - \Lambda_t\,(1 - \xi^{\mathrm{D}}_{N-1})]R^{\mathrm{D}}_{t,N_a-1}, \tag{S15}$$

$$I_{t+1,0} = (1-p)\Lambda_t \sum_a^{N_a} \left[(1 - \xi^{\mathrm{D}}_a)R^{\mathrm{D}}_{t,a} + (1 - \xi^{\mathrm{O}}_a)R^{\mathrm{O}}_{t,a}\right], \tag{S16}$$

$$I_{t+1,i+1} = [1 - \gamma_i]I_{t,i}, \qquad \text{for } i \in [\![1, \dots, N_i - 1]\!], \tag{S17}$$

$$I_{t+1,N_i} = [1 - \gamma_{N_i}]R_{t,N_i} + [1 - \gamma_{N_i-1}]I_{t,N_i-1}, \tag{S18}$$

$$J_{t+1,0} = p\Lambda_t \sum_a^{N_a} \left[(1 - \xi^{\mathrm{D}}_a)R^{\mathrm{D}}_{t,a} + (1 - \xi^{\mathrm{O}}_a)R^{\mathrm{O}}_{t,a}\right], \tag{S19}$$

$$J_{t+1,i+1} = [1 - \zeta_i]J_{t,i}, \qquad \text{for } i \in [\![1, \dots, N_i - 1]\!], \tag{S20}$$

$$J_{t+1,N_i} = [1 - \zeta_{N_i}]J_{t,N_i} + [1 - \zeta_{N_i-1}]J_{t,N_{i-1}}, \tag{S21}$$

$$H_{t+1,0} = \sum_i^{N_i} \zeta_i J_{t,i}, \tag{S22}$$

$$H_{t+1,h+1} = [1 - \eta_i]H_{t,h} \qquad \text{for } h \in [\![1, \dots, N_h - 1]\!], \tag{S23}$$

$$H_{t+1,N_h} = [1 - \eta_{N_h}]H_{t,N_h} + [1 - \zeta_{N_h-1}]H_{t,N_{h-1}}, \tag{S24}$$

$$R^{\mathrm{O}}_{t+1,0} = \sum_i^{N_i} \gamma_i I_{t,i} + \sum_h^{N_h} \eta_h H_{t,h}, \tag{S25}$$

$$R^{\mathrm{O}}_{t+1,a+1} = [1 - \Lambda_t\,(1 - \xi^{\mathrm{O}}_a)]R^{\mathrm{O}}_{t,a} \qquad \text{for } a \in [\![1, \dots, N_a - 1]\!], \tag{S26}$$

$$R^{\mathrm{O}}_{t+1,N_a} = R^{\mathrm{O}}_{t,N_a} + [1 - \Lambda_t\,(1 - \xi^{\mathrm{O}}_{N-1})]R^{\mathrm{O}}_{t,N_a-1}, \tag{S27}$$

$$\text{with } \Lambda_t := \frac{1}{1 + \left(\mathcal{R}_0 c_t \sum_{i=0} \omega_i \left[I_{i,t-i} + J_{i,t-i}\right]\right)^{-1}}. \tag{S28}$$

In this system, we define the following quantities:

- ξ^{D}_a as the decrease in susceptibility due to non-Omicron induced immunity acquired a days ago benefit against Omicron,
- p as the probability of being hospitalised,
- ξ^{O}_a as the decrease in susceptibility individuals previously infected with the Omicron strain a days ago benefit against Omicron,

- γ_i the probability of clearance i days following the infection,
- ζ_i the probability of hospitalisation i days following the infection,
- η_h the probability of leaving the hospital h days following admission.

Similarly, we implemented the polarised model using the following set of equations:

$$R^{\mathrm{D}}_{t+1,0} = 0, \tag{S29}$$

$$R^{\mathrm{D}}_{t+1,a+1} = [1-\sigma^{\mathrm{D}}_a]R^{\mathrm{D}}_{t,a} \qquad \text{, for } a \in [\![1,\dots,N_a-1]\!], \tag{S30}$$

$$R^{\mathrm{D}}_{t+1,N_a} = [1-\sigma^{\mathrm{D}}_{N_a}]R_{t,N_a} + [1-\sigma^{\mathrm{D}}_{N_a-1}]I_{t,N_a-1}, \tag{S31}$$

$$S^{\mathrm{D}}_{t+1} = (1-\Lambda_t)S^{\mathrm{D}}_t + \sum_a^{N_a} \sigma^{\mathrm{D}}_a R^{\mathrm{D}}_{t,a}, \tag{S32}$$

$$I_{t+1,0} = (1-p)\Lambda_t\left[S^{\mathrm{D}}_t + S^{\mathrm{O}}_t\right], \tag{S33}$$

$$I_{t+1,i+1} = [1-\gamma_i]I_{t,i}, \qquad \text{for } i \in [\![1,\dots,N_i-1]\!], \tag{S34}$$

$$I_{t+1,N_i} = [1-\gamma_{N_i}]R_{t,N_i} + [1-\gamma_{N_i-1}]I_{t,N_i-1}, \tag{S35}$$

$$J_{t+1,0} = p\Lambda_t\left[S^{\mathrm{D}}_t + S^{\mathrm{O}}_t\right], \tag{S36}$$

$$J_{t+1,i+1} = [1-\zeta_i]J_{t,i}, \qquad \text{for } i \in [\![1,\dots,N_i-1]\!], \tag{S37}$$

$$J_{t+1,N_i} = [1-\zeta_{N_i}]J_{t,N_i} + [1-\zeta_{N_i-1}]J_{t,N_{i-1}}, \tag{S38}$$

$$H_{t+1,0} = \sum_i^{N_i} \zeta_i J_{t,i}, \tag{S39}$$

$$H_{t+1,h+1} = [1-\eta_i]H_{t,h} \qquad \text{for } h \in [\![1,\dots,N_h-1]\!], \tag{S40}$$

$$H_{t+1,N_h} = [1-\eta_{N_h}]H_{t,N_h} + [1-\zeta_{N_h-1}]H_{t,N_{h-1}}, \tag{S41}$$

$$R^{\mathrm{O}}_{t+1,0} = \sum_i^{N_i} \gamma_i I_{t,i} + \sum_h^{N_h} \eta_h H_{t,h}, \tag{S42}$$

$$R^{\mathrm{O}}_{t+1,a+1} = [1-\sigma^{\mathrm{O}}_a]R^{\mathrm{O}}_{t,a} \qquad \text{for } a \in [\![1,\dots,N_a-1]\!], \tag{S43}$$

$$R^{\mathrm{O}}_{t+1,N_a} = [1-\sigma^{\mathrm{O}}_{N_a}]R_{t,N_a} + [1-\sigma^{\mathrm{O}}_{N_a-1}]R_{t,N_a-1}, \tag{S44}$$

$$S^{\mathrm{O}}_{t+1} = (1-\Lambda_t)S^{\mathrm{O}}_t + \sum_a^{N_a} \sigma^{\mathrm{O}}_a R^{\mathrm{O}}_{t,a}, \tag{S45}$$

$$\text{with } \Lambda_t := \frac{1}{1+\left(\mathcal{R}_0 c_t \sum_{i=0} \omega_i \left[I_{i,t-i} + J_{i,t-i}\right]\right)^{-1}}. \tag{S46}$$

In this system, we define the following quantities:

- σ^{D}_a as the clearance of non-Omicron derived immunity against Omicron,

- p as the probability of being hospitalised,
- σ_a^O as the clearance of the Omicron-derived immunity against Omicron
- γ_i the probability of clearance i days following the infection,
- ζ_i the probability of hospitalisation i days following the infection,
- η_h the probability of leaving the hospital h days following admission.

E Model parametrization

Delay from infection to hospital admission

We parameterised the delay between infection and hospital admission using Salje *et al.* [2020]. In their model, they considered an ODE-based mechanistic transmission model where the path of hospitalised individuals was

$$S \longrightarrow E_1 \xrightarrow{\gamma_1} E_2 \xrightarrow{\gamma_2} I_1 \xrightarrow{\gamma_3} I_2 \xrightarrow{\gamma_4} H_1,$$

where $\gamma_1, \gamma_2, \gamma_3, \gamma_4$, are the compartment departure rates. The delay follows then the hypoexponential distribution

$$\mathcal{H}\text{ypo}\mathcal{E}\text{xp}(\gamma_1, \gamma_2, \gamma_3, \gamma_4) = \mathcal{H}\text{ypo}\mathcal{E}\text{xp}\left(\frac{1}{4}, 1, \frac{1}{3}, \frac{1}{3.5}\right).$$

To simplify our model parametrisation, we discard the hypoexponential distribution by fitting (using `fitdistrplus` package) a close Gamma distribution:

$$\mathcal{G}\text{amma}(\text{SHAPE} : 3.75, \text{ RATE} : 0.33).$$

Delay from infection to recovery (when not hospitalised)

Following the same logic, still from Salje *et al.* [2020], the delay from infection to recovery (entry in the R compartment) for mild infections is

$$S \longrightarrow E_1 \xrightarrow{\gamma_1} E_2 \xrightarrow{\gamma_2} I \xrightarrow{\gamma_3} R,$$

where $\gamma_1, \gamma_2, \gamma_3$ are equal to 1/4, 1, 1/3. This delay follows then the distribution

$$\mathcal{H}\text{ypo}\mathcal{E}\text{xp}\left(\frac{1}{4}, 1, \frac{1}{3}\right).$$

The closest convenient distribution is

$$\mathcal{G}\text{amma}(\textsc{shape}: 2.62,\ \textsc{rate}: 0.33).$$

Delay from hospitalisation to recovery (when not in ICU)

From Salje *et al.* [2020], the time spent hospitalised (when not admitted to the intensive care unit) is

$$\mathcal{G}\text{amma}(\textsc{shape}: 2,\ \textsc{rate}: g^{out}).$$

The parameter value for g^{out} was not available so we assume a value of 0.5, that would lead to a mean hospital stay of four days. Note, this is not important in our study as we focus on hospital admissions.

Omicron-induced hospitalisations

In the daily admission hospital data, we do not know the VOC that originated the infection that led to hospitalisation. We assumed the number of hospitalised due to Omicron (in the early stages) was given by

$$\begin{aligned}\text{Omicron-induced hospitalisations on day } d := {} & \text{Hospitalisations on day } d \times \\ & \text{Omicron VOC frequency on day } (d-10).\end{aligned}$$

The Omciron VOC frequency comes from Sofonea, Roquebert, *et al.* [2022]. The impact of this modification is available on Figure B.

Omicron basic reproduction number

We acknowledge that the notion of basic reproduction number ($\mathcal{R}_0$) does not exist for the Omicron VOC as this number is only defined in an entirely susceptible population and the Omicron VOC emerged two years after the start of the pandemic where all populations across the world had built up (partial) immunity. The parameter $\mathcal{R}_0$ appearing in the force of infection formula is then purely theoretical and represents the Omicron $\mathcal{R}_0$ if Omicron had been introduced in an entirely susceptible population. This is done only for numerical reasons, as it is used to scale the transmission rates for them to have the right order of magnitude. We

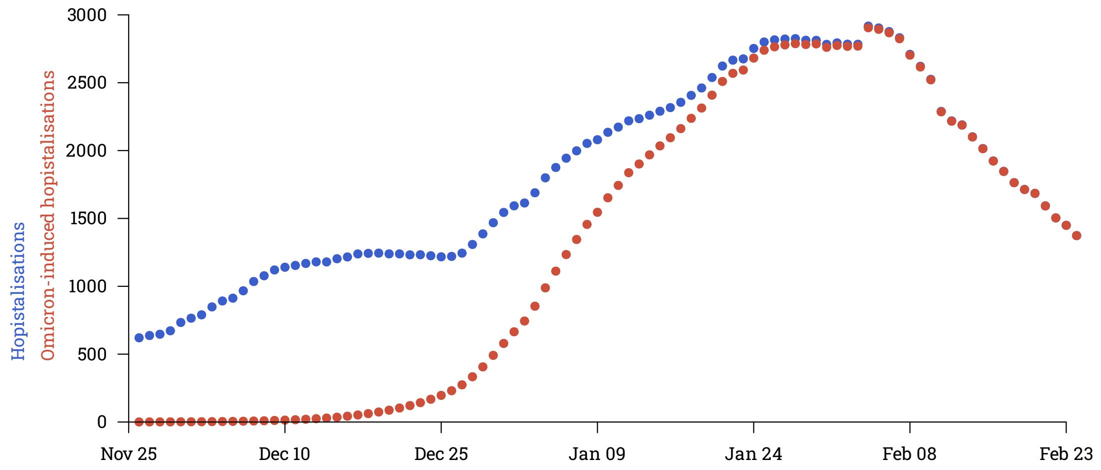

FIGURE B: Correction (in red) applied to hospital admissions (in blue) to disentangle the contribution to the hospitalisations of the Omicron from the other VOCs.

set up an Omicron $\mathcal{R}_0$ of 3, but note that any other values would have caused the model to fit other values for the transmission rates and would not have affected the overall goodness of fit.

Generation time

UKHSA [2022] provided an empirical non-parametric estimation of serial interval for the Omicron BA.2 strain. We used it as the generation strain for the Omicron strains in our model. However, we fitted a $\mathcal{G}$amma(SHAPE : 1.67, SCALE : 1.94) distribution, which is more convenient from a practical point of view.

Immunity effectiveness

STEIN *et al.* [2023] provided estimations of the temporal immunity effectiveness over time at the time of the Omicron invasion. Particularly, they estimated the immunity response against the circulating strains (*i.e.,* immunity waning) and that conferred against the Omicron BA.1 & BA.2 strains (*i.e.*, immune escape).

As you may observe on Figure C, we simplified the trend with linear decay. Furthermore, we assume that initial protection starts at 1 (fully protected) — allowing to start below 1 (some individuals immediately susceptible again) would have require us to re-write the model equations in the the polarised model.

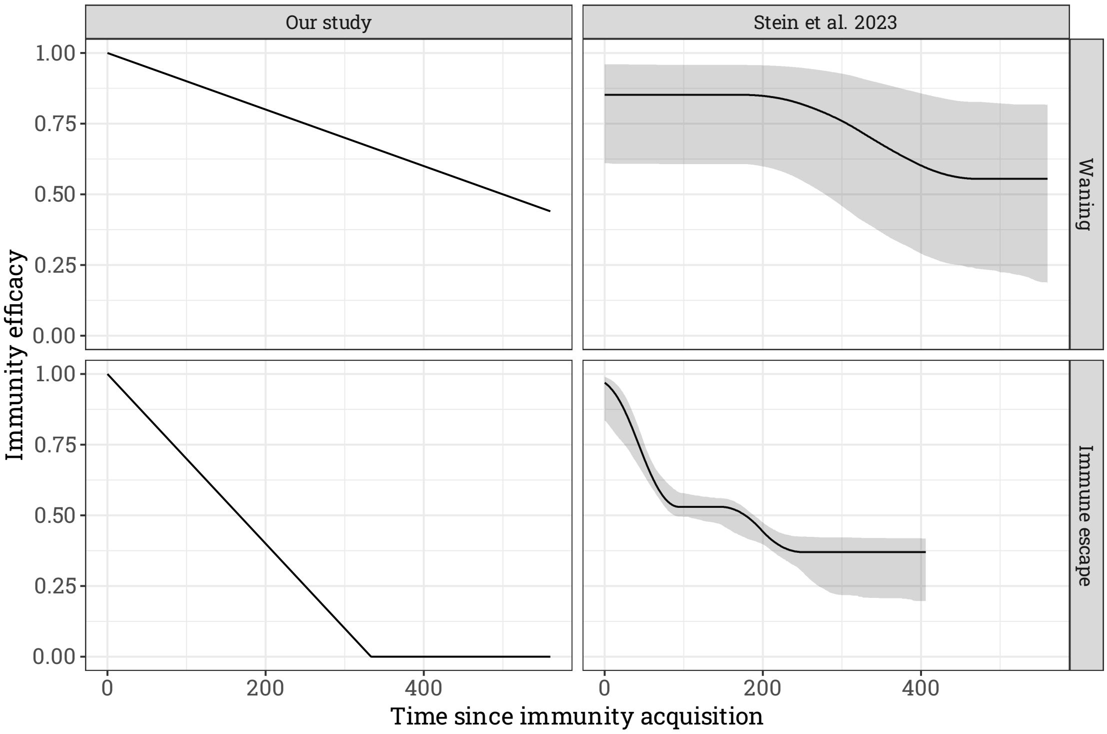

FIGURE C: Comparison on the immunity implemented in our models versus the estimates provided by STEIN *et al.* [2023].

F Bayesian fitting procedure

We used a Bayesian fitting procedure based on KAMIYA *et al.* [2023] using `Stan` [STAN DEVELOPMENT TEAM 2024] (where they also used a non-Markovian discrete-time model of SARS-CoV-2) to reproduce the daily count of new hospital admissions. Our model is, however, simpler with less fitted parameters, as we include only the initial proportion of infected individuals (I_0), the probability of being hospitalised (p) and the transmission rates (c_t). As in KAMIYA *et al.* [2023], we modelled hospital admissions with a log-normal distribution (and standard deviation parameter σ_h).

Priors

We assumed a normal-distributed prior for I_0 (restricted to $\mathbf{R}_+$), as we assumed a high probability to be I_0 somewhere near 0 (in a normalised population size). We have

$$I_0 \sim \mathcal{N}(0.01, 0.01), \qquad \text{with } I_0 \in \mathbf{R}_+.$$

We also assumed a normal-distributed prior for p (restricted to $\mathbf{R}_+$) as this probability is below 0.02. We have

$$p \sim \mathcal{N}(0.01, 0.01) \qquad \text{with } p \in \mathbf{R}_+.$$

Note in both cases these priors are considered as weakly informative as the posterior standard deviations are respectively $\sigma_{I_0} = 1.710^{-5}$ and $\sigma_p = 3.410^{-4}$.

The transmission rates, c_t, were not fitted weekly as in Kamiya *et al.* [2023], as we have more weeks (our modelled period lasts 432 days). We considered a 20-day time window for each transmission rate (meaning a transmission rate is the same for twenty consecutive days instead of a week) leading to a total of 22 transmission rates. We assumed priors normally distributed (and restricted to $\mathbf{R}_+ \setminus \{0\}$) around unity. A transmission rate of one implies the absence of non-pharmaceutical interventions (NPIs) for the reproduction number chosen ($\mathcal{R}_0^{\text{o}} = 3$) and the transmission advantage over the Delta strain comes solely from the immune evasion properties. Note that we do not know either the true $\mathcal{R}_0^{\text{o}}$ (see above) or the NPIs implemented (see Ganser *et al.* [2024] for NPIs intensity earlier in the French SARS-CoV-2 epidemic) as they interfere strongly with the immune effectiveness relative to Omicron. To summarise, we considered

$$c_i \sim \mathcal{N}(1, \sigma_e) \qquad \text{with } c_i \in \ (0, \infty) \text{ for all } i \in \ \{1, \dots, 22\} \text{ and } \sigma_e \sim \mathcal{N}(0, 1).$$

MCMC chains

For each model, we ran four MCMC chains with 2000 iterations each including the warmup of 1000 iterations.

Quality of fit assessments

The model consisted of 24 parameters and two hyper-parameters. We confirmed over 400 effective samples per chain and ensured convergence of independent chains (all $\hat{R}$ values are comprised between 0.98 and 1.02).

Intervals construction

The intervals displayed in Figure 3A are constructed with the 0.025 and 0.975 quantiles for the daily hospital admissions trajectories that were produced with posterior distributions.

G Sensitivity analysis

G.1 Initial immunity sensitivity

By default, we initialised the initial compartment R^D (recovered or vaccinated against Delta VOC or previous strains, or previous vaccination) with a uniform distribution. However, this assumption also influences the initial level of immunity efficacy among the population, which may affect the overall dynamics and results. We performed a sensitivity analysis on this parameter by assuming other initial distributions rather than uniform: one with a mode at 200, one with a mode at 350 and one with a mode at 500. The results are available on Figure D.

Both assumptions generally accommodate the early transmission rates to reproduce the empirical dynamics with similar fitted transmission rates (past the first two months) and consistently fitted the probability of hospitalisation. There is one exception with the polarised model and an initialisation with an early mode (here at 200 days) where the early estimated transmission rates are much higher and the probability of hospitalisation is also estimated with lesser precision. This can be explained by the fact there are fewer susceptible infected individuals early on – when infected individuals reach 1% of the total population, there are only 31% susceptible individuals where it reached 66% with the uniform initialisation.

G.2 Transmission rates sensitivity

We assumed a timespan of 20 days for the transmission rates. However, a concern could be that with enough flexibility on the transmission rates could be fitted any dynamics by overfitting the estimated transmission rates to reproduce the observed dynamics. The difficulty lies in the fact that true transmission rates are unobservable and likely fluctuate over time due to various factors, necessitating the use of time-varying transmission rates. For example, the non-pharmaceutical interventions (NPI) were lifted in March 2023 in France, where the mask-wearing in public spaces was no longer mandatory. Second, factors such as the weather are known to influence the transmission rates [Ma *et al.* 2021]. Third, the Omicron sub-lineages that kept appearing are not accounted for in our model and are smoothed within the changes in the transmission rates.

To ensure our 20-days transmission rates are not simply artificial by-products, we propose

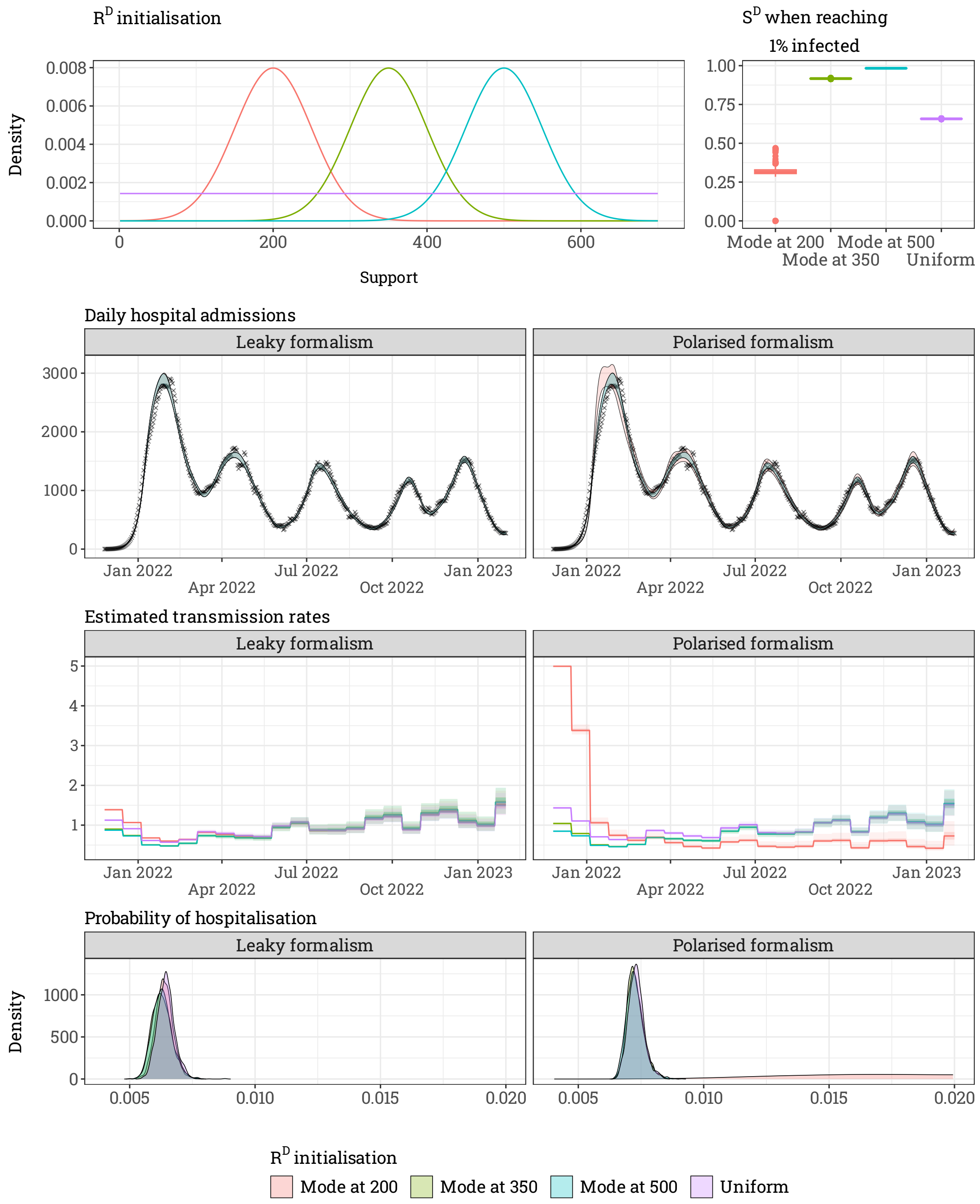

FIGURE D: **Sensitivity analysis on the initial immunity.** First line: left panel show the uniform distribution (our default) in purple and the alternatives tested. Right panel show the proportion of susceptible individuals in the polarised formalism when infected individuals reach 1% of the total population. Second, third and fourth lines show respectively the fitted hospital admissions, the fitted transmission rates and the fitted probability of hospitalisation.

a sensitivity analysis where we reduce the timespan window to 10 days and 1 day (daily transmission rate). The results are shown on Figure E. All models are able to fit the daily hospital admissions dynamic, with a similar probability of hospitalisation. However, the daily transmission rate is subject to a highly variability, with a larger uncertainty, suggesting an overfitting as no NPIs, weather or behavioural drastic changes would be likely to happen on such short scale. On the other hand, we may observe some trends and variations that can be explained (or at least correlated with some events) on the estimations for the 10- and 20-day timespan transmission rate estimations. For instance, an increase in March 2022, that corresponded to the drop in mask-wearing obligations or a decrease between July 2022 and September 2022 which corresponds to the two-months summer holidays.

That being said, we performed an out-of-sample validation of the transmission rates compared to the Google Mobility Data (GDM) available during the same period. GDM time series were reported relative to a baseline pre-COVID era, which corresponds to a period not modelled at this period. To overcome this issue, we only look at the relative magnitude of each GDM times series compared to the relative magnitude of the estimated transmission rates on the matching time periods (GDM data availability and the Omicron period). We define the relative magnitude RM for a times series y_t as

$$RM(t) = \frac{y(t)}{\text{mean}\left((y_t)_t\right)}. \tag{S47}$$

The results are available on Figure F (note the GDM time series have been smoothed out to increase readability – using a roll mean of 7 day). Our findings generally indicate that our estimated transmission rates are less subject to changes than the observed GDM data, which is consistent with the notion that we are not overfitting the transmission rates in our model.

H Model selection and model projections

This study aims to highlight the differences between a leaky immunity and a polarised one at a conceptual level. However, since we fit our models on data, we may also inform this choice with a model selection based on the WAIC. Based on this criterion, the leaky model is selected (difference in expected log posterior density: -31.3, differences in standard errors: 11.2). We used the `loo` package to compute these differences.

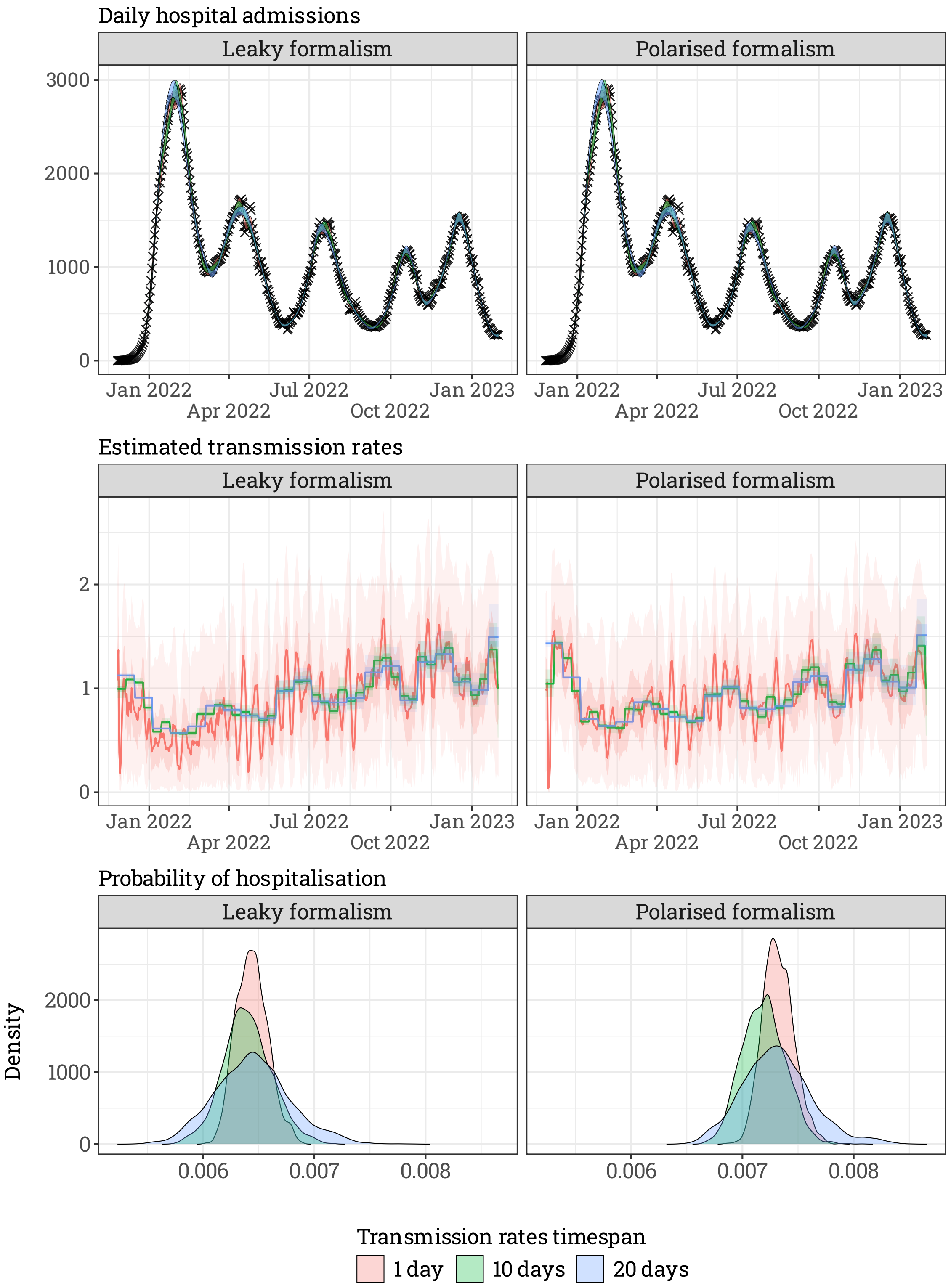

FIGURE E: Transmission rates timespan sensitivity analysis.

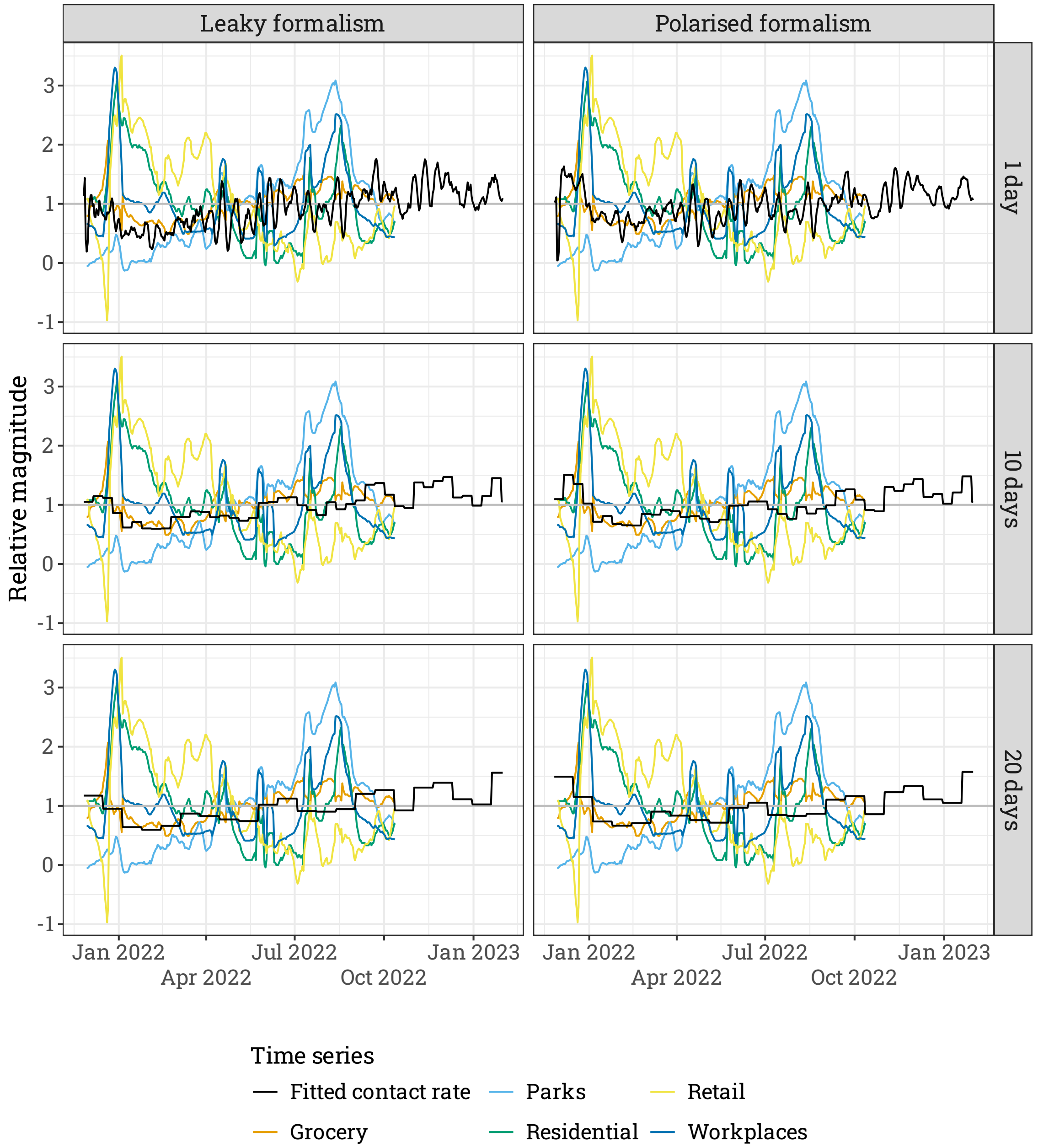

FIGURE F: Transmission rates out-of-sample validation. The black line corresponds to the relative magnitude of our fitted transmission rates, while the colour lines are the relative magnitudes of GDM time series.

Another possibility to illustrate the differences in behaviour is to show their respective projective abilities. To do so, we fitted each model on n days and pursued the dynamics for 30 supplementary days. Note the models are not designed with projections in mind, and the last fitted contact rate was kept for the 30 projections days. We performed this experiment 3 times, with $n \in \{80, 100, 120\}$. It corresponds to three scenarios where the real hospital admissions dynamics behave differently: a monotonous decrease, a sharp switch between a decrease and a sudden increase, and a monotonous increase. The results are shown in Figure G.

Overall, the leaky model has a better median fit (80-day and 120-day scenarios) but a wider uncertainty. Note for the 100-day scenarios (that correspond in reality to the Omicron BA.2 epidemic wave), both models did not predict the reprisal. This is because the data before projection time followed a neat decline, inducing a precise value of the last fitted contact rate.

I Hybrid immunity

We propose to study the mathematical behaviour of a simple model with hybrid immunity. The idea of a hybrid model is that immunity could be either leaky (with a proportion p) or polarised (with a proportion $1-p$). This is mostly done to explore the mathematical behaviour, as the biological processes behind this assumption are not straightforward.

There is a subtlety in whether this proportion acts at the infection level or if it depends on the individuals (Figure HA). In the latter case, the endemic equilibrium depends on the proportion of individuals with a leaky immunity. When the dichotomy between leaky and polarised acts is at the infection level (meaning that p infections end up with a leaky immunity upon clearance), the equilibrium tends to the polarised one. This is because the polarised immunity is longer than the leaky one.

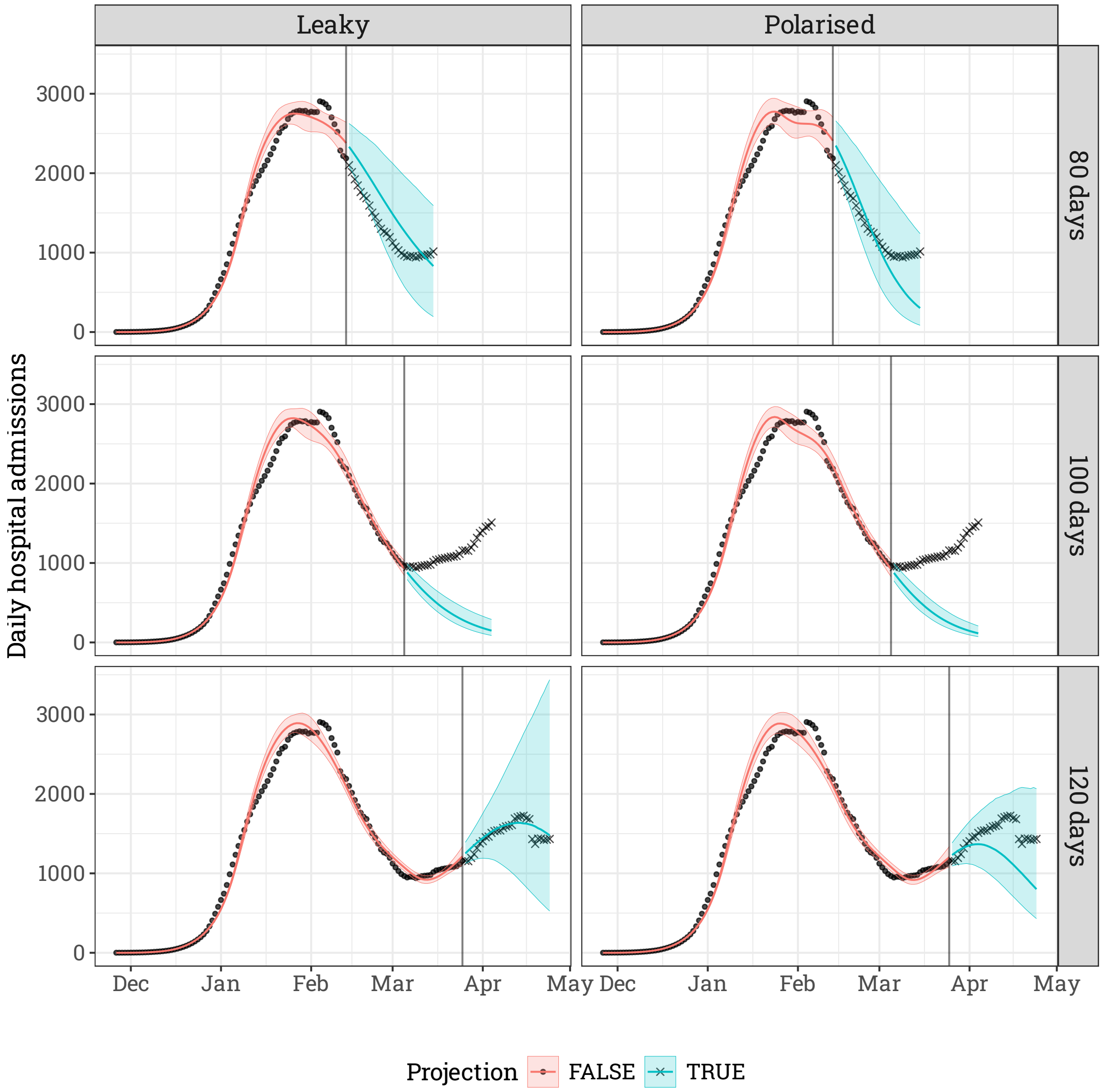

Figure G: **Model projections up to 30 days in three different settings.** The red area corresponds to the fitted on the real data (black dots), and the blue corresponds to model projections. The black crosses represent the real data not used to calibrate the model.

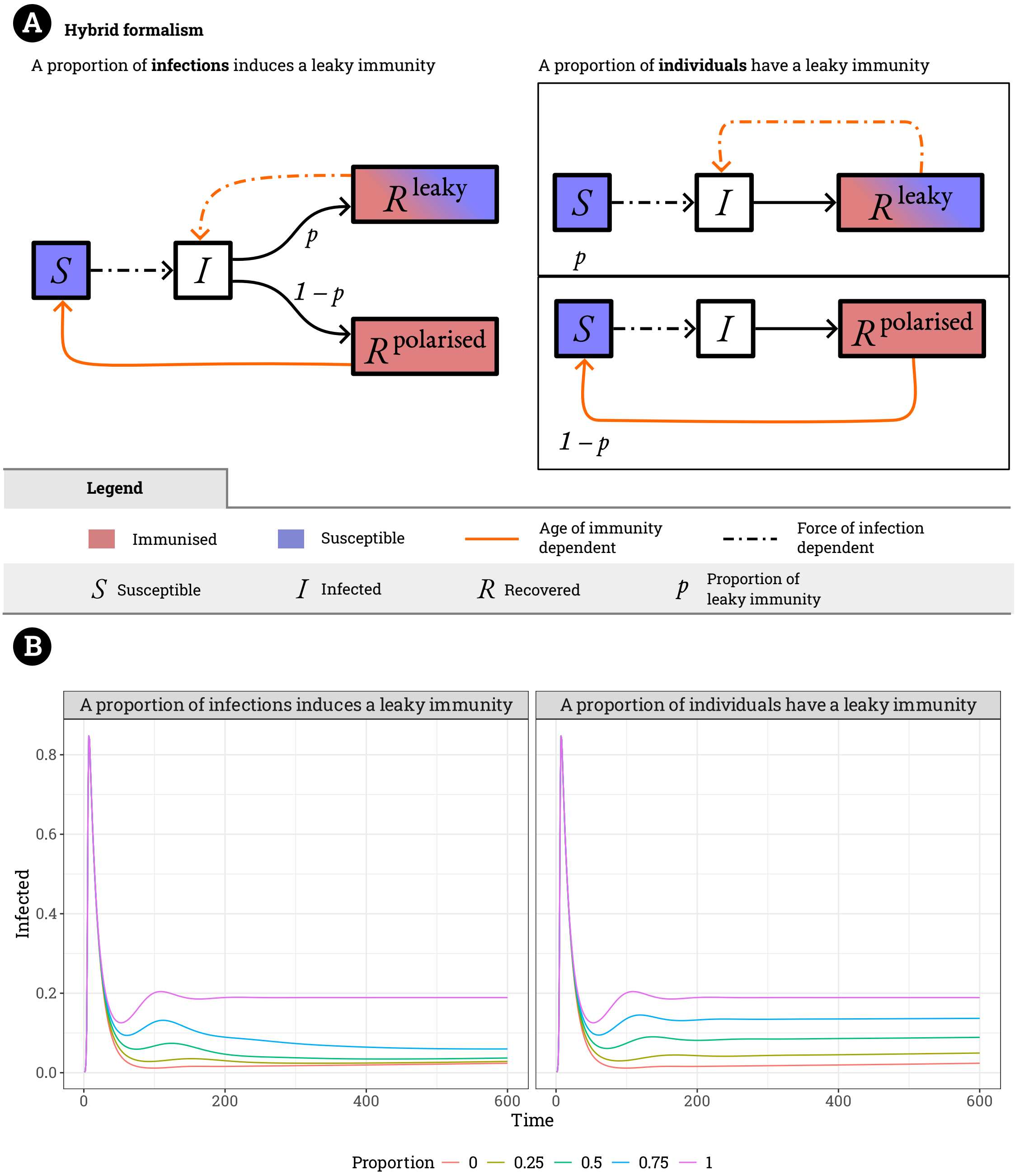

Figure H: **Hybrid immunity model. A.** This represents at which level the dichotomy between leaky and polarised may happen: at the infection level (left side) or the individual level (right) side. **B.** Dynamics of the presented models, with $\mathcal{R}_0 = 4$, a half-life infection duration of 14 days, and a decreasing immunity efficacy described in Section E.

References

Blythe, S. P. and Anderson, R. M. (1988). "Distributed Incubation and Infectious Periods in Models of the Transmission Dynamics of the Human Immunodeficiency Virus (HIV)". *Mathematical Medicine and Biology*. doi: 10.1093/imammb/5.1.1.

Diekmann, O. *et al.* (2021). "The discrete-time Kermack–McKendrick model: A versatile and computationally attractive framework for modeling epidemics". *Proceedings of the National Academy of Sciences*. doi: 10.1073/pnas.2106332118.

Elie, B., Selinger, C., and Alizon, S. (2022). "The source of individual heterogeneity shapes infectious disease outbreaks". *Proceedings of the Royal Society B*. doi: 10.1098/rspb.2022.0232.

Ganser, I. *et al.* (2024). "Estimating the population effectiveness of interventions against COVID-19 in France: A modelling study". *Epidemics*. doi: 10.1016/j.epidem.2024.100744.

Hethcote, H. W., Stech, H. W., and Van Den Driessche, P. (1981). "Nonlinear Oscillations in Epidemic Models". *SIAM Journal on Applied Mathematics*. doi: 10.1137/0140001.

Kamiya, T. *et al.* (2023). "Estimating time-dependent contact: a multi-strain epidemiological model of SARS-CoV-2 on the island of Ireland". *Global Epidemiology*. doi: 10.1016/j.gloepi.2023.100111.

Keeling, M. J. and Rohani, P. (2008). *Modeling Infectious Diseases in Humans and Animals*. Princeton University Press. doi: 10.2307/j.ctvcm4gk0.

Kermack, W. O. and McKendrick, A. G. (1927). "A contribution to the mathematical theory of epidemics". *Proceedings of the Royal Society of London. Series A, Containing Papers of a Mathematical and Physical Character*. doi: 10.1098/rspa.1927.0118.

— (1932). "Contributions to the mathematical theory of epidemics. II. —The problem of endemicity". *Proceedings of the Royal Society of London. Series A, Containing Papers of a Mathematical and Physical Character*. doi: 10.1098/rspa.1932.0171.

Lloyd, A. L. (2001). "Destabilization of epidemic models with the inclusion of realistic distributions of infectious periods". *Proceedings of the Royal Society of London. Series B: Biological Sciences*. doi: 10.1098/rspb.2001.1599.

Ma, Y. *et al.* (2021). "Role of meteorological factors in the transmission of SARS-CoV-2 in the United States". *Nature Communications*. doi: 10.1038/s41467-021-23866-7.

Salje, H. *et al.* (2020). "Estimating the burden of SARS-CoV-2 in France". *Science*.

Sofonea, M. T., Reyné, B., *et al.* (2021). “Memory is key in capturing COVID-19 epidemiological dynamics”. *Epidemics*. doi: 10.1016/j.epidem.2021.100459.

Sofonea, M. T., Roquebert, B., *et al.* (2022). “Analyzing and Modeling the Spread of SARS-CoV-2 Omicron Lineages BA.1 and BA.2, France, September 2021–February 2022 - Volume 28, Number 7—July 2022 - Emerging Infectious Diseases journal - CDC”. *Emerging Infectious Diseases*. doi: 10.3201/eid2807.220033.

Stan Development Team (2024). *The Stan Core Library*.

Stein, C. *et al.* (2023). “Past SARS-CoV-2 infection protection against re-infection: a systematic review and meta-analysis”. *The Lancet*. doi: 10.1016/S0140-6736(22)02465-5.

Svensson, Å. (2007). “A note on generation times in epidemic models”. *Mathematical Biosciences*. doi: 10.1016/j.mbs.2006.10.010.

UKHSA (2022). *SARS-CoV-2 variants of concern and variants under investigation report 36*.

Wilson, E. B. and Burke, M. H. (1942). “The Epidemic Curve”. *Proceedings of the National Academy of Sciences*. doi: 10.1073/pnas.28.9.361.

Yorke, J. A. (2006). “Selected topics in differential delay equations”. *Japan-United States Seminar on Ordinary Differential and Functional Equations: Held in Kyoto/Japan, September 6–11, 1971*. Springer.
