# Supplementary material for: Leaky or polarised immunity: Non-Markovian modelling highlights the impact of immune memory assumptions
Source: PLoS Comput Biol. 2025 Aug 19;21(8):e1013399. doi: 10.1371/journal.pcbi.1013399 (PMC12407549; doi:10.1371/journal.pcbi.1013399)
Supplement: Attachment — Submitted filename: Review Leaky vs Polarised.pdf [file pcbi.1013399.s003.pdf]

Review of "Leaky or polarised immunity: non-Markovian modelling highlights the impact of immune memory assumptions"

In short:

- Main idea of leaky versus polarised formalism is well-explained, implications of choosing one over the other also well-executed
- Model explained at some depth, but inconsistent treatment of the actual model
- Question remains: how can one know when to apply which formalism? How strong is the impact of choosing the same model parametrisation?

Discussion

Does a person, after an infection, stay recovered until, with some probability, becoming susceptible, or does the protection of a cleared infection wane over time? The authors of the paper at hand consider this question by comparing the implementations of the "leaky" versus the "polarised" immunity formalism. Viewed in terms of the classical SIR model, in either case for both formalisms, after a cleared infection, the path goes to a staggered R compartment, namely a chain of compartments representing each one (discrete) time step having elapsed since the cleared infection (the staggered chain is the non-Markovian part, right?). The difference between the leaky and the polarized model is now the following: for the leaky formalism, individuals may move from any subcompartment of R directly back to the infected compartment, but for the polarized model, there is an added step between, the susceptible compartment, which acts as a sort of "gatekeeper". In essence, as this reviewer understands it, in the leaky model, the S compartment only feeds into the infected compartments but does not receive any individuals from the recovered compartments; on the other hand, in the polarised model, any individual passes from a subcompartment of R first through S before re-entering an infected compartment.

Both Figure 1 and Figure 2 are quite helpful in explaining the difference between the two formalisms, though in both figures, it may thus be beneficial to the uninitiated to display the R compartment not as $R_1 ... R_N$ in one compartment but draw them as separate boxes in a chain (as, I believe, was done in previous work of the authors for such chains of subcompartments). Moreover, it is confusing in Figure 1C that the leaky formalism keeps the susceptible compartment, but then in Figure 2, the leaky formalism is depicted without a susceptible compartment. Indeed, even in the subsequent equations describing the leaky model, there sometimes is an S compartment (which, by Equation (S8), page 31, only ever depletes), and sometimes not (e.g. in lines 201-204 on page 13, or in F, Equations (S16)-(S31) on page 35).

Some questions on the maths: why is the S compartment staggered into a chain in Equations (5)-(9), too, using *s* as the time unit recording how long ago the immunity was lost? Could the authors comment on why they included this in the polarised model in Equations (5)-(9), but did not include this visually in either Figure 1 or 2, or in the model equations in (S32)-(S49) (also, please note you erroneously called both sets of model equations in Appendix F "leaky")? Moreover, the recurrence systems in Equations (2)-(4) and in Equations (5)-(9) are confusing, since you give $R_{0,0}$ and $R_{t+1, a+1}$, but not $R_{t,0}$, which gives the false impression that *t* and *a* are not distinct. Lastly, please indicate clearly what you mean by non-Markovian early in the main text and not just in the Appendix; it needn't be in mathematical terms, but it aids in understanding why you choose this model. On a different note, could you comment on how the chain length N of the subcompartments of R was chosen? Is it chosen in some distributional sense? What impact does this choice have on the model output, and why?

All this to say that the paper explains the main idea of the leaky versus the polarised formalism quite well and manages to strike a balance between explaining the intricacies versus giving the intuition behind the concept. However, this paper strikes me as a methods paper, but the method could be more consistently explained, both in the main part and in the Appendix, and many figures that help in discerning the differences between the leaky and the polarised immunity formalism could be moved from the Appendix to the main part. For a methods paper, more of the actual method should be discussed in the paper, if not using equations, then at least discussing Figures S1, S2 and S5. In addition, Figure S5 is not clear to me; can you be more specific what you mean by "the models' outputs for the Omicron epidemic waves fit" (lines 760-761)? What is the output precisely you use for the Sobol' method here? In general, it would be appreciated if you could explain the sensitivity analysis in more detail.

Additionally, it isn't quite clear how the leaky and polarised model would differ if you did not fix some parameters to be the same for both; can you comment on this? And could you comment also on your assertion that applying the polarised model results in "an *increased error* in the estimate of the numbers of recovered individual[s]"? You explain well how on a short-term scale, the difference between the two models is negligible, but how can you know that on longer time scales, the error lies with the polarised one? And could this have to do with the model parametrisation being the same?

In summary, it is a nice paper with a clear thread running through it and a worthwhile model. The paper may benefit from a reread for typos and grammar, specifically in the Appendix. Moreover, there are some additional, minor points below.

Minor points:

- Typos and grammar mistakes, such as "fo" instead of "of" on page 10, line 128, "french" instead of "French" in the main text, and then more in the Appendix, among them "have not include", (p.25, l.443), "be taken with cautious" (p.25, l.445), "fit an high" (p.25, l.445), the sentence spanning lines 463-468. For example, Section B.1, parts of Sections B.2 and C would all benefit and thus gain legibility
- For different forms of the function f(a), have you also tried a piecewise defined function with a constant plateau somewhere?
- The proof for more reinfections in the leaky formalism than in the polarised one seems to be correct, yet the step from t_1 to t_2 follows because $q_p(t_1) = q_l(t_1)$, but for subsequent steps it should follow because $q_p(t_m) \leq q_l(t_m)$ for $m \geq 2$. But how then can we deduce the inequality (S15) for $m \geq 2$? (i.e. you say "[we] could continue further showing that $q_p(t_3) \leq q_l(t_3)$ following the same logic") I think the induction works but is not as immediate as it was for the induction base case.
- It is somewhat counterintuitive that for the many recurrence systems you give in both the main part and in the Appendix, any equation is given as e.g. $R_{t+1,a+1}$, which, as I understood it, means however that *a* denotes which subcompartment in the chain we are in, and *t* denotes time, i.e. of what this subcompartment is a function of. The fact that both *t* and *a* are subscripts is a bit odd if you have a fixed number of subcompartments.
