# Supplementary material for: Leaky or polarised immunity: Non-Markovian modelling highlights the impact of immune memory assumptions
Source: PLoS Comput Biol. 2025 Aug 19;21(8):e1013399. doi: 10.1371/journal.pcbi.1013399 (PMC12407549; doi:10.1371/journal.pcbi.1013399)
Supplement: Attachment — Submitted filename: REBUTTAL.pdf [file pcbi.1013399.s004.pdf]

Response to reviewers

The authors deeply thank the three reviewers for their careful proofreading and constructive comments, which we believe increased the quality of our manuscript. All three reviewers asked for methodological clarifications in the main text and fewer details kept in the appendix, which led to a restructuring of our manuscript. In this revised version of our manuscript, we tried to account for all of the remarks made and our main changes include:

- A more self-sufficient main text.
- Changes in methodology in the first part, which corrects a reasoning error and simplifies a lot of the formalism.
- Replacement of the Sobol-based sensitivity analysis (which was not well adapted) by sensitivity analyses on the initialisation of the R^D compartment and on the transmission rates.
- More discussion about the limits of the approach.

We list hereafter our answers (in black) to reviewers' remarks (in blue).

Reviewer #1

> The manuscript investigates an important and interesting question in infectious disease modeling: How does the specific formalism used to describe immunity waning influence model dynamics/outcomes. The authors compare the leaky formalism (recovered individuals become more and more susceptible to reinfection as time since last infection/vaccination increases) and the polarized formalism (recovered individuals are fully immune but become fully susceptible over time). These two formalisms are frequently used in infectious disease models. For COVID-19 and many other diseases, the former formalism appears to be more useful but polarized immunity cannot be completely discounted. The authors display a great understanding of mathematical epidemiology and nicely motivate the choice of modeling framework.

Thank you for the accurate summary of our study and this positive appreciation!

> That being said, the most interesting discussions and answers related to the research question are in the various appendices (I am not sure why). The large number of appendices negatively affects the flow of the paper.

Our first intent was to focus specifically on the conceptual differences regarding the immunity assumptions (leaky vs. polarised) and to leave in the Appendix as many technical details as possible, including the non-Markovian parts. We re-structured the manuscript to make the main text more self-sufficient by adding many details to guide the readers.

> Moreover, this manuscript suffers from poor English. While I realize that the authors are French and likely lack a native English speaker, a lot of the typos and grammar issues could have been prevented by the use of same English language software, e.g. Microsoft Word. Further, wording is at times confusing and the brevity of many explanations (i.e., lack of details) makes it hard to follow along.

We apologise for the poor English and clearly realise it could have greatly benefited from further corrections before getting released.

> Most importantly, I have severe concerns about the research findings. 1. The claim "the time between reinfections is always shorter with a leaky formalism" appears to be obvious given the way the authors implemented the two formalisms, specifically given the assumption of constant force of infection and in the absence of disease dynamics. See major comment 1 below. 2. The COVID-19 results appear to be largely confounded by assumptions made about initial conditions, which are not described anywhere, so I can only assume that the authors assumed everyone was recently recovered with 0 days since last immunity event at the onset of the Omicron waves, clearly a wrong assumption that affects the comparison between the leaky and polarized formalism. See major comment 2 below.

We thank you for stressing this point and reply below after the major comment.

> **Major comments:**

> **1.** The authors should provide more details in the results section, clarifying the assumptions underlying the finding "the time between reinfections is always shorter with a leaky formalism", specifically focusing on the validity of the strong assumption of a constant force of infection.

We simplified the assumptions in the main text. Our initial derivation, as pointed out by Reviewer #3, was flawed. The revised version relies on fewer assumptions — eliminating compartment models in favour of a simple induction (lines 85–95 and Appendix A.1). Regarding the constant force of infection, we felt it would be more insightful for the readers to start with a toy model before investigating a more applied case. However, we agree that the details provided were too scarce for this example to be useful as it was.

> Upon reading the Methods in detail I came to the conclusion that the results shown in Fig S1 in support of the claim "the time between reinfections is always shorter with a leaky formalism" are in a way a "self-fulfilling prophecy/circular conclusion": The authors use the exact same function f in both formalisms. The polarized formalism assumes individuals that "lose immunity" become first susceptible, at which point they are susceptible to infection. The leaky formalism assumes individuals are always vulnerable to infection but a decreased rate. In other words, individuals are moved into the S and I compartment at the same rate, parametrized by the function f, in the polarized and leaky formalism, respectively. Naturally, this means reinfections occur earlier in the leaky formalism. We don't need a model to obtain this result, as long as the force of infection is assumed to be constant and the same for the two formalisms.

At first glance, it might seem obvious that the time before reinfection is shorter in the leaky model than in the polarized one, as individuals may be reinfected without first returning to S. However, following this line of reasoning, we should expect only a shift in the times before reinfection between the polarized and leaky models? As suggested by Reviewer #3, we added a piecewise function (see Figure 2, bottom panel), which we believe is a good illustration of the non-trivial phenomenon occurring here. Indeed, although the same function f is used in both models, it can still display important differences. We hope the new examples better highlight the non-trivial aspects behind this behaviour.

Note: the authors are aware of this, and derive this result, somewhat confusingly and with many assumptions, in Appendix E.

We included this part in the main text, clarified the assumptions and corrected the error in the proof mentioned by Reviewer #3. We also got rid of some unnecessary assumptions. Overall, we hope that this simpler version of the framework is easier to grasp now.

2. Figure 2 is very nice and helpful. It would be nice if the authors could add further details to the main text (Lines 91-94), specifically related to the initial values used in the models: at the beginning of the omicron wave (when the authors assume everyone has attained some immunity through infection or vaccination), how is the population distributed in terms of age of immunity in $R^D_{t=0,a}$, and what proportion, in the polarized formalism, starts in S^D? This strongly affects the outcomes! I could not find any information regarding this.

We thank you for highlighting this lack of justification and apologize for the lack of clarity. In the polarised formalism, S^D was set to 0 at start $t = 0$. However, with initialisation of $R^D(t = 0, a)$ (uniform by default), many individuals were distributed at an age a where the loss of immunity rate was quite high, leading to the early production of a large number of susceptible individuals. By the time infected individuals reached 1% of the total population there was roughly 65% individuals entirely susceptible. We now make our assumption explicit in the model section and discuss it more thoroughly in the discussion. Following Reviewer #2's suggestion, we also performed a sensitivity analysis on this specific assumption (Appendix G.1), which now allows us to give additional details about the S^D compartment for the different initialisation settings explored.

Side note: Not everyone will have been infected with Delta. I would change "Last infection by Delta VOC" to something like "Immunity due to non-Omicron infection or vaccination". See and modify also lines 220-225.

Thank you for the suggestion. We made the change.

3. The 95% posterior trajectories intervals look identical for the leaky and the polarized formalism. Are the authors sure that this is correct and not a copy paste

> error? Given differences in the number of reinfections between the formalisms, I would expect to see differences in hospital admissions as well.

We double checked our results and can confirm that the posterior trajectories intervals are identical. This can be explained because we specifically fit the number of daily hospital admissions, and, since both models can reproduce the dynamics, their trajectories appear very similar. However, there are small numerical differences. We now discuss the fitting procedure more thoroughly, especially the 20-days span for the contact rates parameters, an aspect for which we now include a sensitivity analysis (Appendix G.2).

> Also, the total number of reinfections appears to be almost double in the leaky vs all-or-nothing formalism (3B) but the differences in daily infections (3C) are much smaller. This is strange, or am I missing something?

The model parameters are fitted to reproduce the observed number of daily hospital admissions. The number of reinfections is computed as the infected individuals who were previously infected by Omicron. There are very few at the beginning, and it increases over time as many people become infected at least once in both formalism. Since the number of reinfections is not equal to the cumulative number of infections (but reinfections), there is no reason for them to show the same order of magnitude. This difference is a consequence of our result that the time between two reinfections is shorter in the leaky formalism, inducing the difference in the cumulative number of reinfections is expected to increase over time.

> Lastly, the authors should explain in the Results what exactly the "probability of hospitalization" refers to.

It is the probability of being hospitalised following an infection. It corresponds to parameter p_h in the model, and it's the probability of entering compartment J (infected individuals who will require a hospitalisation) upon infection (in the new version, in the Methods section, in the second paragraph).

> **4.** The appendix should not include new results. All results and supplementary figures should be referred to in the main manuscript, e.g., the global sensitivity analyses.

We followed your suggestion and also improved our sensitivity analyses. More precisely, we removed the Sobol-based sensitivity analysis, which was less relevant given our main question and which we did not bring much material for discussion. We replaced it with sensitivity analyses on aspects that are more in line with the results, especially the importance of the initial level of immunity and of transmission rate estimates. We hope this makes the revised version of our manuscript more focused and easier to understand.

> **Minor comments:**
>
> L17: It would be nice if the authors could expand a little on "conservative assumptions", either rewrite or add some more info.

We rewrote this sentence to provide more details.

> L44/45: The sentence for the leaky vaccine is wrong. The decrease in susceptible is of size x, not $1 - x$. I.e., if $x = 80\%$, then the susceptibility of vaccinated/recovered individuals is $1 - x = 20\%$ compared to that of non-vaccinated/non-recovered people.

Yes, you are right, it is a decrease of x, and hence a multiplicative factor of $1 - x$. We corrected the mistake. Thank you for the careful reading.

> Fig 1C: R_N in black on dark blue cannot be read.

We changed the colour in the figure.

> L86: awkward wording: "hardly remains generalisable" - is it generalizable or not? are the multiple reinfections the issue or the dynamical (i.e., non-constant force of infection) setting?

We clarified the formulation. Thank you for the suggestion.

> L155: a bimodal distribution of immune efficacy. It is unclear how this ties in with using antibody levels as correlates of protection. I hope the authors can clarify this in the text by rewriting/expanding this paragraph.

We rewrote this paragraph to explain how the heterogeneity in the antibodies distributions across individuals may highlight a wide heterogeneity of patterns of immunity efficacy.

> L504-514: I do not get the purpose of this paragraph. Using the strict definition of non-Markovian, yes, an SEIR model can be seen as non-Markovian. However, the way an SEIR model is typically implemented (using a fixed transition rate from E to I) it is indeed Markovian. The authors should clarify their point. Especially, because according to lines 528-530, the standard SEIR model would be Markovian even according to the authors.

Our point was only to underline that the definition of non-Markovian could be subject to discussion and say that, in a way, most models could fall in that category. We aim to specify our usage of the term non-Markovian specifically states we record explicitly the time since infection or recovery and we are not just adding some heterogeneity in the residence times.

> 616 "proportion of individualS that HAVE become ..." - it's important to

We corrected the mistake. Thanks!

> Appendix G: It would be nice to see plots/statistics that exhibit the goodness of fit between the hypo-exponential and Gamma distributions.

We show the goodness of fit for the distributions in Appendix G and below in Figure 1. We did not include this graph in the manuscript as we think this departs from the main topic of this study.

> Sobol's indices should be followed by a reference. Also, the information provided related to the sensitivity analysis is insufficient to reproduce the results.

As already mentioned, we removed this part and replaced it by a more focused sensitivity analysis.

> Language:
>
> - L4: "and" not "or"verifies
> - L14/15: "made the ...", grammar

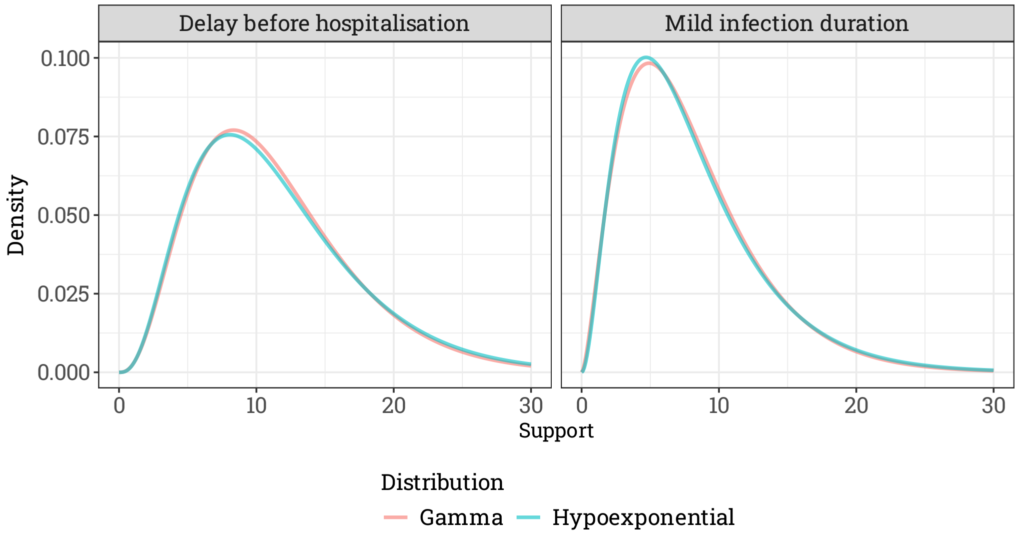

FIGURE 1: Comparison between Gamma and hypoexponential distributions.

- L25: p missing: susceptible
- L26/27: "It is the case of the first studies" - strange wording, not sure what exactly this means.
- L45/46: if x in [0,1], get rid of % signs everywhere here. Or let x in [0,100] but that is the worse choice.
- L47: delete to in "or to natural"
- Fig 1 caption: simplest, not most simple
- L64: assesS
- L63: strange wording "This is partic- ularly the case of the study by"
- L65: strange wording "as it should be studied further"
- l74: "using simple modelS" or "using A simple model"
- L128: "can fo unnoticed"???
- L147: strange wording: "never discontinued to assess "
- l170: strange use of "verifies": we introduce the following function that verifies for all a
- Fig 2 caption: the only difference ... IS the following:
- L212: witH
- L215: not sure what this is supposed to mean: "We implement the two types of immunity described to SARS-CoV-2 incidence data"
- L222: to illustrate, not illustrated
- L433: grammar: "in every cases"

- l433: I would add the word partial: “we assume partial immunity to last... “
- L438: strange wording: “which is an order of magnitude observed quite frequently “
- L480: replace “will” by “ability”?
- L482: strange wording: “It will manifest itself through a wide variety of approaches over time”. E.g., why is future tense used?
- l489: replace “may allow using of” by “enables the use of the”
- L552: “implying a manual Euler scheme” is confusing. How about (if this is what the authors mean): “requiring the implementation of a manual Euler scheme”
- l552: replace “way more easy” by “easier”
- L558: awkward: “the choice goes to the modeller who will choose”
- L566: become, no s
- L570: “become infected becomeS”
- 577: “SAME” not smae
- 581: noticeD
- 585: would may ??
- 586: “The used Equation” - which equation? Why capitalize equation?
- 587: grammar: “as it do no involve”
- 607: re-infected, other places: reinfected - be consistent
- 609: I would add: “fully recovered (I.E., FULLY IMMUNE) before being ...”
- 623: strange wording: “move also on the age of immunity support as time flies”
- 642: t_2
- 654: delete “we made”
- 655: Model equations
- 677: from of

This list is by no means exclusive. Any English language program, e.g. Microsoft Word, would have caught >50% of these errors.

We apologise for the poor English. We corrected the errors listed and polished the text more carefully in the revised version.

Reviewer #2

I would like to begin by thanking the authors for their thorough and thought-provoking work on "Leaky or polarised immunity: non-Markovian modelling highlights the impact of immune memory assumptions." I appreciate the time and effort that went into developing the non-Markovian model, performing the numerical experiments, and analyzing different immunity assumptions. In my opinion, this study addresses an important question related to COVID-19 modeling: whether immunity is better captured by a "leaky" or a "polarized" mechanism. Below, I offer my comments and suggestions, which I hope will be useful as you revise the manuscript.

Thank you very much for all your constructive and helpful comments and remarks.

1. Major Points

1.1 Single population group (no age or comorbidity stratification)
In my opinion, one of the key limitations of the current model is the absence of any population stratification by age or risk factors. Given that COVID-19 severity, hospitalization rates, and reinfection risks vary significantly with age and comorbidities, modeling the entire population as one homogeneous group may bias the results.
— The average risk of hospitalization or severe outcomes could be quite different from reality if a large proportion of hospitalized individuals actually belong to higher-risk subgroups. In my opinion, this limitation could affect the interpretation of the results regarding reinfection rates and hospitalization patterns.
— I believe it would strengthen the paper to include at least two or three age/risk strata. If doing so is not feasible in the current version, you might consider discussing thoroughly how homogeneous assumptions could skew your results.

These suggestions echo some of our previous work on COVID-19, in which we explicitly include an age structure of the population, in addition to the age of the infection and show the importance of the contact matrix between the ages on the epidemic dynamics (Reyné et al.

2022). We could easily have reused the same model to have more detailed dynamics. However, the main goal of this article is to compare the leaky and the polarised formalisms, and we think such a high-level of detail would be detrimental to the main objective for several reasons.

As we showed in Reyné et al. (2022), this stratification introduces a lot of uncertainty regarding the contact age matrix, which is poorly known. Another difficulty is that the age of newly hospitalised individuals is not publicly available in France, meaning that it would not be possible to fit the epidemic dynamics by age groups. Finally, we do not think that the absence of age class introduces a major bias in our results as the attack rate is roughly the same across age groups (see e.g. French public health agency (Santé Publique France) report [1] and its Figure 1 that represents the seroprevalence across age-groups in the first year of the pandemic). In the revised version, we now mention our earlier results using a non-Markovian model with age structure and explain why we used a simpler version here.

> **1.2** Assumption of "fully immunized population" before Omicron
>
> In the section "Modelling the initial waves of the Omicron variant," the text suggests that everyone was already immunized (by either Delta infection or vaccination) prior to Omicron's emergence. In my opinion, this may be overly simplistic because, in reality, there was still a fraction of people without prior vaccination or infection, and vaccines were not 100% effective against Delta.
>
> — This assumption could distort the susceptible and reinfection dynamics during the Omicron wave, leading to a mismatch with real-world situations.
>
> — I think it would be important to justify this assumption more comprehensively or conduct a sensitivity analysis. Discussing the robustness of your conclusions under different levels of pre-Omicron immunity would also be helpful.

This a very valid point that we neglected in the first version and was also raised by Reviewer #1: we did not write down nor discuss the initialisation of the immunity against pre-Omicron existing strains. We now propose a sensitivity analysis on how we initialise it (Appendix G.1) and explain it further in the discussion.

Please also note that the case of individuals without prior immunisation is taken into account

[1] https://www.santepubliquefrance.fr/etudes-et-enquetes/covid-19-etudes-pour-suivre-la-part-de-la-population-infectee-par-le-sars-cov-2-en-france

when the initialisation includes individuals with a decreased in susceptibility of 0 % (in the leaky model) or $S^D(t = 0) > 0$ in the polarised one. However, this does again raise the question of the homogeneity assumption, as no contact matrix is implemented in this case.

> **1.3** Strict comparison between "leaky" and "polarized," with no hybrid models
> In my view, comparing two extreme forms of immunity—leaky (partial protection for all) vs. polarized (some are fully protected, others are not at all)—is certainly insightful. However, there is empirical evidence (e.g., for COVID-19) that real populations might exhibit hybrid responses, with gradations between these extremes.
> — By focusing only on the two polar extremes, the numerical results might overstate the differences. In my opinion, many real-world scenarios likely fall in between.
> — I suggest, at minimum, a deeper discussion of possible hybrid scenarios and the biological rationale for focusing on these two extremes. Highlight that leaky and polarized are presented as limit cases—if you do not model hybrid immunities, I believe it is necessary to justify clearly why these extremes suffice for the study.

The reviewer is perfectly correct in stating that these two formalisms are extreme cases of a continuum, and we now present them as such. This point is stated more clearly in the revised version of the Introduction and is now discussed more in the context of the immune boosting theory, described in Park *et al.* [2024] and applied in a recent preprint by Cellès *et al.* [2025] on pertussis. We now also mention the recent work by Graham & Regöes [2024], who discuss the impact of the inoculum sizes on the infection duration.

> **1.4** A posteriori calibration of contact rate without predictive comparison
> The manuscript mentions a Bayesian fitting procedure in which the contact rate is adjusted every 20 days to match hospitalization data. In my opinion, this approach may obscure structural uncertainties in the model: with sufficient flexibility in the contact rate, almost any epidemic curve might be replicated, making it hard to distinguish the impact of leaky vs. polarized immunity.

— This could diminish the predictive power of the model, especially if you cannot show how well the model performs in periods not used in the calibration.
— I would recommend including some predictive checks or out-of-sample validation to ensure that the model's ability to match hospitalization curves is not merely a byproduct of repeatedly adjusting the contact rate. A stronger discussion of how these calibration intervals might affect the main conclusions would also be valuable.

We agree with this remark, although we want to stress that we do not think there exists a perfect method to ensure the absence of any kind of over-fitting. In order to present a more rigorous approach, we now perform an out-of-sample validation using the Google Mobility data, which is, to our knowledge, the only exogenous proxy for contact rate easily accessible. We then compare the data available on the overlapping, that is, from Nov. 26th 2021 (our start) up to Oct. 15th 2022 (Google end). The results display that the relative magnitude of our contact rate is lower than the Google Mobility data relative magnitude in the same period, suggesting that the "byproduct" is not artificial.
Furthermore, we also performed a sensitivity analysis by fitting the model assuming transmission rates definition windows of 10 days, as well as a daily transmission rates (Appendix G.2). The latter exhibit a noisy pattern that we do not retrieve in the 10-day or 20-day transmission rate window. Note that in our model transmission rates increase towards the end, because of the lack of new Omicron sub-strains and related immune evasion properties implementation.

1.5 Lack of direct comparison with empirical data
— The article references certain observational studies (e.g., Lind et al. [2023]) that suggest leaky immunity in specific outbreak settings. In my opinion, however, the manuscript does not explicitly confront the model's outputs with real-world data or attempt to empirically differentiate which immunity regime is more plausible.
— If the goal is to elucidate leaky vs. polarized immunity in practice, I believe some additional validation or at least a discussion of possible empirical markers for each regime could greatly enhance the paper's relevance.
— In my view, I encourage you to propose ways to validate leaky vs. polarized

immunity assumptions beyond the single observational reference. It would help readers connect the model results with real data more concretely.

The reviewer's suggestion is interesting as, indeed, a transmission model could potentially be used to infer the type of immunity (leaky *vs.* polarised) using incidence-like data (but without any immunity-related data). This may very well raise identifiability issues and largely depend on the quality of the data available, which is why it is beyond the point of this work. Our goal here is to show that different assumptions regarding immunity, which reflect biological differences that can be (and have already been) estimated using field data, strongly impact epidemiological dynamics; a question that is usually overlooked. We do agree that existing data is limited and the recent literature on that subject is quite scarce. One possible explanation is that it is difficult to elaborate a satisfactory correlate of protection from real-world data. This difficulty to measure immunity at an individual level or population level makes it difficult to propose a simple rule to choose between the two formalisms. We now discuss this problem towards the end of the manuscript.

2. Minor Points

2.1 Potential confusion around terminology

— Example The phrase "basic reproduction number (R0) of Omicron" is used in the modeling section. Strictly speaking, R0 is defined in a fully susceptible population, so calling it an R0 for Omicron can be misconstrued if the population is not fully susceptible.

In my opinion, it would be clearer to label this parameter an "effective reproduction rate" or clarify that it is a hypothetical scaling factor.

This was detailed in Appendix G of the original version and we moved it to the main text when discussing "Omicron-era baseline reproduction number".

2.2 Functions for waning and immune escape

— The manuscript refers to multiple functional forms for waning immunity and immune escape in appendices, but the main text does not go into much detail.

I think providing more biological or empirical justification for each function in

the main text would improve clarity. Even just a short paragraph summarizing the rationale (linearity, sigmoid decay, etc.) would help.

Our intent with the different shapes for waning immunity was mainly to show that no matter the mathematical form, the time was always shorter with the leaky formalism. We did not link it to any biological justification (*e.g.* a particular shape for a particular pathogen), but one could be that no matter the true biological form of the waning immunity, from a mathematical point of view, it would still lead to shorter time between reinfections with the leaky formalism.

2.3 No differentiation between vaccine-derived and infection-derived immunity — The model lumps all "immunized" individuals together, without distinguishing vaccine-induced vs. infection-induced immunity. In reality, these might have different waning kinetics and protective profiles.
In my opinion, the manuscript should acknowledge and briefly discuss this limitation. If further stratification is impractical, explaining why it is not crucial for your primary conclusions would be beneficial.

Thank you for pointing this It is true only the age of immunity affects the immunity efficacy. We now briefly discuss these limitations in the Discussion.

2.4 Absence of mortality in the model
The model flow is infection → hospitalization → recovery, with no explicit compartment or rate for COVID-19 fatalities.

Even though COVID-19 mortality may be relatively small compared to total infections, in my opinion, it could influence long-term dynamics. If mortality is omitted, a brief statement about its potential impact would be appropriate.

We think it might have marginally diminished the number of reinfection (individuals that died could not be reinfected). But we do not expect changes in the conclusions of our manuscript. We now acknowledge this limitation in the Discussion.

3. Overall Recommendation: Major Revisions

When I weigh these points, I conclude that there are enough substantive issues—particularly around population stratification, the assumption of universal pre-Omicron immunity, the dichotomy between leaky vs. polarized, and the a posteriori calibration of contact rates—that, in my opinion, this manuscript would benefit from major revisions.

— I want to emphasize that I genuinely appreciate the sophisticated non-Markovian framework, the focus on exploring different immunity mechanisms, and the numerical experiments presented. I believe the paper addresses a timely question and has strong potential.

Thanks for the very kind words.

— In my opinion, the main text should discuss more thoroughly the key assumptions (e.g., homogeneous population, no mortality, universal immunization before Omicron) and their potential impact on the interpretation of the results. If it is not feasible to change the model structure now, a robust justification and discussion of limitations would still greatly strengthen the manuscript.

As you will see in the revised version, we tried to take into account all the suggestions you made. In particular, we now clearly discuss each limiting assumption and propose new sensitivity analyses to strengthen some of our claims.

4. Suggestions to Improve Clarity and Organization

1. I realize that in detailed modeling papers, it is common to place extensive derivations and supplementary analyses in the appendices. However, in my opinion, some critical justifications (for instance, the choice of waning functions, contact-rate calibration details) should be outlined more explicitly in the main text so that the reader does not need to constantly switch to the supplementary material.

We have restructured the manuscript to include more details in the main text. We hope that this makes the revised version easier to read.

2. I notice that some figure legends are quite long, almost repeating sections of the discussion or methods. In my opinion, figure legends should provide all essential information to interpret each figure but should not become overly redundant. Perhaps you could move lengthy methodological explanations to the main text or the appendix, keeping figure legends more concise but still self-contained.

We tried to shorten the figure legends as much as possible.

3. In my perspective, the manuscript might flow more smoothly if references to modeling assumptions and their justifications were systematically included in the main "Methods" and "Results" sections, with clear pointers to the appendices for further mathematical details.
— For instance, "We use a flexible Bayesian approach to recalibrate the contact rate every 20 days (see Appendix X for details)."
— This approach would keep the main text succinct but still let readers see the rationale without flipping back and forth too often.

To improve the clarity of the Appendix, we restructured it so that it better matches the structure of the main text.

Overall, I believe that incorporating these adjustments and clarifications—especially regarding stratification, justification of assumptions, discussion of hybrid immunity, and more robust calibration checks—will significantly strengthen the manuscript. In my opinion, once these major points are addressed, the paper will be in a much better position to communicate its valuable findings to the readership of PLOS Computational Biology.

I thank the authors again for their work and for the opportunity to review this submission. I hope my comments will be taken in the constructive spirit intended, and I look forward to seeing a revised version of this interesting study.

Thank you again for all those criticisms, which we found very relevant and constructive, as well as for the kind words.

Reviewer #3

In short:

· Main idea of leaky versus polarised formalism is well-explained, implications of choosing one over the other also well-executed
· Model explained at some depth, but inconsistent treatment of the actual model
· Question remains: how can one know when to apply which formalism? How strong is the impact of choosing the same model parametrisation?

Discussion

Does a person, after an infection, stay recovered until, with some probability, becoming susceptible, or does the protection of a cleared infection wane over time? The authors of the paper at hand consider this question by comparing the implementations of the "leaky" versus the "polarised" immunity formalism. Viewed in terms of the classical SIR model, in either case for both formalisms, after a cleared infection, the path goes to a staggered R compartment, namely a chain of compartments representing each one (discrete) time step having elapsed since the cleared infection (the staggered chain is the non-Markovian part, right?).

In the revised version of the manuscript, we redid the figures to improve their clarity. We also now include more details about non-Markovian models in the main text.

The difference between the leaky and the polarized model is now the following: for the leaky formalism, individuals may move from any subcompartment of R directly back to the infected compartment, but for the polarized model, there is an added step between, the susceptible compartment, which acts as a sort of "gatekeeper". In essence, as this reviewer understands it, in the leaky model, the S compartment only feeds into the infected compartments but does not receive any individuals from the recovered compartments; on the other hand, in the polarised model, any individual passes from a subcompartment of R first through S before re-entering an infected compartment.

> Both Figure 1 and Figure 2 are quite helpful in explaining the difference between the two formalisms, though in both figures, it may thus be beneficial to the uninitiated to display the R compartment not as $R_1 ... R_N$ in one compartment but draw them as separate boxes in a chain (as, I believe, was done in previous work of the authors for such chains of subcompartments).

Thank you for the suggestion. We updated Figure 1 accordingly.

> Moreover, it is confusing in Figure 1C that the leaky formalism keeps the susceptible compartment, but then in Figure 2, the leaky formalism is depicted without a susceptible compartment.

Figure 1C does not refer to any model used in this paper. Its goal is to illustrate how the difference between leaky and polarised immunity could be represented using the very classical *SIR* model. Regarding Figure 2, we assume that everyone gained some immunity (previous infection or vaccination) by the time Omicron arrives (late 2021). That implies the lack of susceptible compartment in the leaky formalism, although some previously recovered individuals might suffer from a waning immunity (depicted with the colour gradient). In the polarised formalism, individuals may completely lose their immunity and hence be entirely susceptible again (hence the susceptible compartment).

> Indeed, even in the subsequent equations describing the leaky model, there sometimes is an S compartment (which, by Equation (S8), page 31, only ever depletes), and sometimes not (e.g. in lines 201-204 on page 13, or in F, Equations (S16)-(S31) on page 35).

There are indeed two cases.

The first one is when we use very simple models (*i.e.*, toy-models), as in Equation (S8). These models do not correspond to any real outbreak, and we may initialise them with an entirely susceptible population. In that case, the susceptible compartment only ever depletes because once susceptible individuals have been infected, they enter into the recovered compartment. But as the immunity becomes 'leaky', individuals may be reinfected (with a probability that depends on the age of immunity). Note this first option was removed (lines

201-204 in the initial version) from the revised version of the manuscript in order to simplify it.

In the second case, which is showed in Figure 2, we propose an application on real data, starting with the first Omicron epidemic waves, two years after the beginning of the pandemic. By that time, we assumed almost all individuals had gained some immunity (either by a previous infection or vaccination), which is why we avoided the term ‘susceptible’ (as entirely susceptible) and replaced it by the term ‘susceptibility’, which may account for different levels of susceptibility. The susceptibility is then set to a given level, and we explore various initialisation scenarios, with individuals being more or less susceptible.

> Some questions on the maths: why is the S compartment staggered into a chain in Equations (5)-(9), too, using s as the time unit recording how long ago the immunity was lost? Could the authors comment on why they included this in the polarised model in Equations (5)-(9), but did not include this visually in either Figure 1 or 2, or in the model equations in (S32)-(S49) (also, please note you erroneously called both sets of model equations in Appendix F “leaky”)?

The reviewer is entirely correct in that we only stated we needed the time spent susceptible in Equations (5)-(9) without explaining why. While in this model, nothing depends on the exact age of the time spent in the susceptible state, we need to record the time spent uninfected, *i.e.* the time spent fully recovered plus the time spent susceptible but not yet reinfected. Conversely, in the model captured by in Equations (S32)-(S49) we do not record the time spent uninfected and, therefore, do not include this age stratification. Note that due to the rewriting of the first part of the manuscript, equations (5)-(9) were removed in the revised version.

> Moreover, the recurrence systems in Equations (2)-(4) and in Equations (5)-(9) are confusing, since you give $R_{0,0}$ and $R_{t+1,a+1}$, but not $R_{t,0}$, which gives the false impression that t and a are not distinct. Lastly, please indicate clearly what you mean by non-Markovian early in the main text and not just in the Appendix; it needn’t be in mathematical terms, but it aids in understanding why you choose this model.

We now provide more details about the advantages of non-Markovian properties directly in the main text (at the beginning of the Model section). We also remove the Equations (1)-(9) that were quite confusing.

> On a different note, could you comment on how the chain length N of the subcompartments of R was chosen? Is it chosen in some distributional sense? What impact does this choice have on the model output, and why?

We choose $N = 700$ to be large enough to guarantee that individuals recovered for N days are entirely susceptible again (*i.e.* the decrease in susceptibility is 0 in the leaky model and all individuals are susceptible again in the polarised one) for the R^D compartment. N was also chosen to be greater than the total number of simulated days to avoid problems for the R^O compartment, as individuals never reached the upper limit. This information is now in the main text (in the Model section). We also performed a sensitivity analysis on the initialisation of R^D (Appendix G.1) to explore the importance on the model output.

> All this to say that the paper explains the main idea of the leaky versus the polarised formalism quite well and manages to strike a balance between explaining the intricacies versus giving the intuition behind the concept. However, this paper strikes me as a methods paper, but the method could be more consistently explained, both in the main part and in the Appendix, and many figures that help in discerning the differences between the leaky and the polarised immunity formalism could be moved from the Appendix to the main part. For a methods paper, more of the actual method should be discussed in the paper, if not using equations, then at least discussing Figures S1, S2 and S5.

As indicated in our reply to the other reviewers, we restructured the manuscript for the main text to be more self-contained. We hope this makes the revised version easier to read.

> In addition, Figure S5 is not clear to me; can you be more specific what you mean by "the models' outputs for the Omicron epidemic waves fit" (lines 760-761)? What is the output precisely you use for the Sobol' method here? In general, it would be appreciated if you could explain the sensitivity analysis in more detail.

We removed the Sobol-based sensitivity analysis because, as pointed out by another reviewer, it was not the most relevant sensitivity analysis to perform. Instead, we now perform sensitivity analyses two particular aspects which are the initialisation of the system and the transmission rate windows.

> Additionally, it isn't quite clear how the leaky and polarised model would differ if you did not fix some parameters to be the same for both; can you comment on this?

We heartily agree and, in fact, in the model, we did not fix the probability of being hospitalised upon infection to answer this specific question. In the end, our model does provide two different estimates in the two models. Our conclusion is that the estimations of the state of the epidemics (or its long term dynamics) may be biased because of the modelling choice regarding immunity.

> And could you comment also on your assertion that applying the polarised model results in "an increased error in the estimate of the numbers of recovered individual[s]"?

This comment described our specific SARS-CoV-2 case, in which we assumed the "true immunity" to be leaky as pledged by Lind *et al.* [2023]. We now dampen it a little bit by acknowledging that the true immunity could be somewhere in the middle, as suggested by Reviewer #2, but the overall interpretation remains the same: if the immunity is mispecified, then some key metrics (*e.g.* the number of recovered individuals) can be biased.

> You explain well how on a short-term scale, the difference between the two models is negligible, but how can you know that on longer time scales, the error lies with the polarised one? And could this have to do with the model parametrisation being the same?

It was probably not right to be adamant with the polarised being completely wrong for the SARS-CoV-2, and the leaky formalism the "true immunity" since the actual data is very limited. We now discuss more carefully how these two formalisms can be seen as extreme limit cases, and how a wide variety of immunity profiles could happen in real life (*e.g.* with the immunity boosting paradigm for instance).

In summary, it is a nice paper with a clear thread running through it and a worthwhile model. The paper may benefit from a reread for typos and grammar, specifically in the Appendix. Moreover, there are some additional, minor points below.

Minor points:

Typos and grammar mistakes, such as "fo" instead of "of" on page 10, line 128, "french" instead of "French" in the main text, and then more in the Appendix, among them "have not include", (p.25, l.443), "be taken with cautious" (p.25, l.445), "fit an high" (p.25, l.445), the sentence spanning lines 463-468. For example, Section B.1, parts of Sections B.2 and C would all benefit and thus gain legibility.

Thank you for your careful reading! We tried to clarify all mistakes and typos

For different forms of the function f(a), have you also tried a piecewise defined function with a constant plateau somewhere?

We now include a function of this kind. Thank you for the suggestion.

The proof for more reinfections in the leaky formalism than in the polarised one seems to be correct, yet the step from t_1 to t_2 follows because $q_p(t_1) = q_\ell(t_1)$, but for subsequent steps it should follow because $q_p(t_m) \leq q_\ell(t_m)$ for $m \geq 2$. But how then can we deduce the inequality (S15) for $m \geq 2$? (i.e. you say "[we] could continue further showing that $q_p(t3) \leq q_\ell(t3)$ following the same logic") I think the induction works but is not as immediate as it was for the induction base case.

Thanks a lot for pointing this out! Indeed, the induction we presented was wrong and we went to hastily about it. As it turns out, there may even exist some time steps t_m where $q_p(t_m) > q_\ell(t_m)$. We corrected the proof and now use the idea of looking at the remaining quantities of uninfected individuals with each formalism instead of increments of newly infected individuals. We hope this provides the same message in a clearer (and more correct) way, with fewer assumptions.

It is somewhat counterintuitive that for the many recurrence systems you give in both the main part and in the Appendix, any equation is given as e.g. $R_{t+1,a+1}$, which, as I understood it, means however that a denotes which subcompartment in the chain we are in, and t denotes time, i.e. of what this subcompartment is a function of. The fact that both t and a are subscripts is a bit odd if you have a fixed number of subcompartments.

In classical ODEs-based models, what is sometimes referred to as the 'chain trick' would make individuals leave R_1 for R_2 at a given rate, and so on, to further compartments R_N. However, with the ODE approach, the duration for which individuals in a compartment such as R_2 have been recovered is not straightforward. In our approach, the index a specifically indicates the time individuals have been recovered for, which allows us to make the immunity efficacy depend on that age. The double indices we use could probably be interpreted with partial differential equations-based models, where compartments may have two time indices (*e.g.* the time t and the age of immunity a), as described in Appendix B.
