# Supplementary material for: Leaky or polarised immunity: Non-Markovian modelling highlights the impact of immune memory assumptions
Source: PLoS Comput Biol. 2025 Aug 19;21(8):e1013399. doi: 10.1371/journal.pcbi.1013399 (PMC12407549; doi:10.1371/journal.pcbi.1013399)
Supplement: Attachment — Submitted filename: Review_PLOS_2_R1.pdf [file pcbi.1013399.s006.pdf]

Review of revised version of "Leaky or polarised immunity: non-Markovian modelling highlights the impact of immune memory assumptions"

It is a pleasure to see that the authors have restructured the exposition of the paper, and that it adds a lot of value in terms of clarity and completeness. It is also commendable that the authors went into detail when answering all the points raised by the reviewers. In my opinion, all comments have been sufficiently addressed, especially the exposition of the methods – in the Appendix, but also the main part –, the sensitivity analysis, and the comparison between the leaky and polarised formalism.

I have a few remaining minor points:

- In line 101, you write "Assuming \tau = 0, we have …". But what you assume is that q_l(0)=q_p(0)=1 at \tau = 0. It should be phrased accordingly, i.e. "We assume q_l(0)=q_p(0)=1," as otherwise your assumption would refer to \tau.
- You write "The number of remaining uninfected individuals …", but it seems as if q_l and q_p are in fact proportions of uninfected individuals? (if q_l(0)=q_p(0)=1)
- On the same note, only if q_l and q_p represent proportions do your calculations imply that q_l(\tau) <= q_p(\tau), as q_l(\tau)<=1 (otherwise your induction step from 1 to 2 doesn't work). You should add a comment on this.
- In the Results section, in Equations (1) and (2), q_l and q_p at time \tau+1 depend on the values of q_l, q_p respectively, and both also on \xi, at time \tau. Yet, in the Appendix in (S1), (S2) etc. you falsely write \xi evaluated at the time step \tau+1.
- In the Appendix on the first page, you have some Equations not yet linked, showing up as ??.

With these comments addressed, I can fully recommend this article for publication.
