# Supplementary material for: Leaky or polarised immunity: Non-Markovian modelling highlights the impact of immune memory assumptions
Source: PLoS Comput Biol. 2025 Aug 19;21(8):e1013399. doi: 10.1371/journal.pcbi.1013399 (PMC12407549; doi:10.1371/journal.pcbi.1013399)
Supplement: Attachment — Submitted filename: REBUTAL_R2.pdf [file pcbi.1013399.s007.pdf]

Response to reviewers

We would like once again to thank all three reviewers for their thoughtful comments. Among the most important changes in this second round, we included in the Supplementary information a 'toy' model illustrating a hybrid approach where individuals in the same population can either benefit from a leaky or a polarised immunity. We also performed a proper WAIC model selection as well as projections at 30 days given different subsets of the daily hospital admissions time series.

We list hereafter our answers (in black) to reviewers' remarks (in blue).

Reviewer #1

> The authors took the comments and concerns from me and other reviewers seriously and the revised manuscript is substantially improved. I just have a few language suggestions.
>
> Language:
> l113: delete "epsilon(tau) where"
> l162: replace "divergences" by "differences"
> l192: "a unique" not "an unique"
>
> Note: all my line number references are based on the marked up manuscript page numbers.

We corrected these typos. Thanks again for the careful reading.

Reviewer #2

> I would like to thank the authors again for their thoughtful revisions and clarifications to the manuscript. In my earlier review, I raised several major points, which I will revisit here in light of the new version. Overall, the manuscript has improved in terms of clarity and explicit acknowledgment of potential

limitations. However, a few substantive issues remain and merit the authors' consideration before final acceptance.

1. Single Population Group (No Age or Comorbidity Stratification)

Original Concern (1.1):

I noted that modeling the entire population as homogeneous may bias results, since COVID-19 severity, hospitalization risks, and reinfection dynamics vary substantially by age and comorbidities.

Authors' Response and Revisions

• The authors now discuss in the text that they assume a single age group and justify that "the attack rate was similar across age groups in France."

• They argue this choice keeps the model simpler, emphasizing the difference between "leaky" vs. "polarized" immunity rather than the heterogeneities of subpopulations.

What Remains

• While the authors do acknowledge this limitation, the paper still provides limited quantification or sensitivity analysis on how much ignoring age/risk structure might skew hospitalization estimates.

• In practice, hospital admissions skew older, so the time to reinfection and probability of severe disease could differ in a multi-strata model. In my opinion, a short statement or additional sensitivity test (even if minimal) could strengthen the argument that "the homogeneous approach does not invalidate the key result."

We now further acknowledge the limitation about the single population group and indicate that it could be an important aspect to account for in future studies (line 164).

Several reasons precluded performing additional analyses to assess the robustness of this assumption. First, this would require age-stratified hospital admissions time series, which are unavailable for France. Using data from another country would then raise the issue about obtaining precise contact matrixes, which are rarely available, highly variable, and bring a lot of variance in the model output (see one of our previous work, Reyné *et al.*, 2022, *MMNP*). Finally, we could use simulated data but then the number of *ad hoc* assumptions would be too

high to achieve robust results and, furthermore, this would require an amount of work which is beyond the scope of this study.

However, we insist on the fact that the attack rate was homogenous across age groups in France [1]. That is why we think our assumption that population-averaged severity-related rates and proportions are stationary holds.

2. Assumption of "Fully Immunized Population" Before Omicron

Original Concern (1.2):

I questioned whether assuming that everyone was immunized by Delta or vaccination before Omicron might distort subsequent dynamics, since a fraction remained non-immune in reality.

Authors' Response and Revisions

• The revised manuscript explicitly includes a sensitivity analysis (see their Appendix G) where the initial "recovered" compartment is populated differently (uniform or modes at different times).

• They note that this helps illustrate that the exact fraction of pre-Omicron immunity does not drastically alter the qualitative difference between leaky and polarized assumptions.

What Remains

• While the new text clarifies the setup, it might still be worth more explicitly acknowledging that a small fraction truly had zero prior protection.

• Overall, the authors' sensitivity checks appear sufficient to show that the key result—shorter time to reinfection under "leaky" vs. "polarized"—is robust under multiple initial distributions.

We now state explicitly in the main text that some individuals are fully susceptible in the beginning for both models (line 282).

3. Strict Comparison Between "Leaky" and "Polarized," With No Hybrid Models

[1] cf. see Figure 1 at `https://www.santepubliquefrance.fr/etudes-et-enquetes/covid-19-etudes-pour-suivre-la-part-de-la-population-infectee-par-le-sars-cov-2-en-france`

Original Concern (1.3):

Real-world immunity likely lies between these two extreme assumptions. I suggested either (a) a numerical exploration of intermediate/hybrid immunity or (b) a deeper discussion justifying the focus on these two extremes.

Authors' Response and Revisions

• The authors add some discussion referencing works on partial or "immune boosting" frameworks (e.g., Park et al. [2023]) and note that these extremes capture two endpoints of imperfect immunity.

What Remains

• Despite the discussion, there is still no direct simulation or parametric test of a "mixed" approach (e.g., 50% fully protected, 50% partially protected).

• I recognize that adding this would expand the study's scope considerably, but even a short numerical experiment or scenario analysis for a "fraction leaky vs. fraction polarized" would be valuable to show whether real data might fall between the extremes.

We added a 'toy' model in the Supplementary information (Section I) to account for this final scenario raised by the reviewer. We observe a continuum of the endemic equilibrium values depending on the proportion of individuals that benefit from a leaky immunity.

Please also note that the biological rationale for such a hybrid assumption, where half the population benefits from a leaky immunity or a polarised one, is perhaps a bit far-fetched. We also performed a fit of the model with a 50−50 % hybrid immunity, that produces intermediate estimations (see Figure 1). We did not include these results in our manuscript (nor in the Supplementary information) because we do not think they bring further insights into the questions of interest.

4. A Posteriori Calibration of Contact Rate Without Predictive Comparison

Original Concern (1.4):

By re-fitting the contact rate in 20-day windows, the model can accommodate many dynamics. I asked for out-of-sample checks or predictive validation to ensure that repeated calibration does not mask fundamental differences between

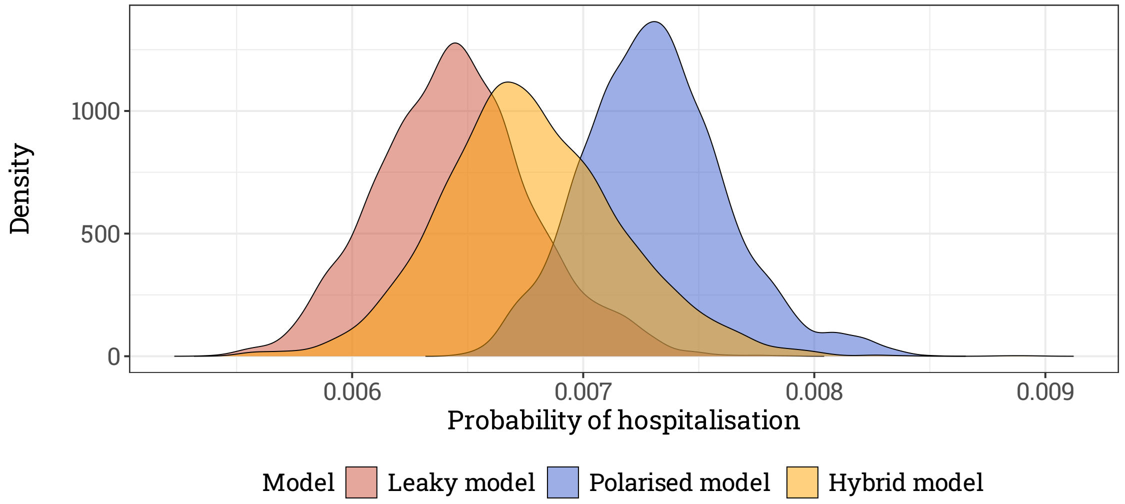

FIGURE 1: Posterior distributions of the hybrid model compared to the leaky and the polarised ones.

immunity assumptions.

Authors' Response and Revisions

• In the supplement, they analyze the sensitivity of results to 10-day vs. 20-day fitting windows, and briefly compare the fitted transmission rate to Google Mobility Data.

• They argue this partly addresses the risk of overfitting because they see some consistency with exogenous mobility trends.

What Remains

• While these additions help, we do not see a strict out-of-sample forecast (fitting up to date T, then predicting T+1 onward). Consequently, the difference in predictive power between leaky vs. polarized remains somewhat unclear.

• If the manuscript's main objective is more conceptual than predictive, this may be acceptable. Nonetheless, clarifying that further predictive validations were not the focus might help.

Thanks to this second round of review, we realised that we did not understand Reviewer #2 meant 'forecasting' and/or 'projections' in the first review round when he spoke of out-of-sample validation'.

Our main objective in this work is more conceptual than predictive. This implies that our

models are not tailored to predict accurately the dynamics.

However, to address the reviewer's concern, we included some projections that may help to further clarify the differences between the leaky and polarised formalisms. More precisely, we fit both models on the first 80, 100, and 120 days and let the dynamics run for 30 more days, with the last fitted contact rate. This corresponds to three different scenarios, where the dynamics are respectively decreasing, decreasing just before a sharp increase of cases, and increasing. It highlights two main patterns: both models are equally bad in the context of a sharp reprisal, and the leaky model has the best median but a wider uncertainty. All the relevant details can be found in Supplementary Information (Section H).

5. Lack of Direct Comparison With Empirical Data

Original Concern (1.5):

I suggested that the authors try to differentiate which immunity regime fits the data better, perhaps via a likelihood-based comparison or referencing more than one observational study.

Authors' Response and Revisions

• They do point to Lind et al. [2023] and note that certain data suggest real SARS-CoV-2 immunity is "leakier" than previously thought.

• The new version also more openly discusses how real data might help identify leaky vs. polarized assumptions in practice.

What Remains

• The paper still does not present, for example, an AIC or WAIC comparison between the two models applied to the same dataset. We only see that both formalisms can reproduce hospitalization curves once contact rates are adjusted.

• A formal model-selection approach might still be out of scope, but at least acknowledging that the two frameworks might be empirically discriminated in longer time series or in controlled cohorts is helpful.

We now perform a formal model selection using the WAIC criterion, which favours the leaky model over the polarised one. We included this result in the same section of the Supplementary information as the projections detailed above (Section H).

Summary of Recommendations

• **Hybrid Immunity:** If feasible, add a brief scenario or appendix section illustrating what happens when a fraction of individuals has "polarized" immunity and another fraction has "leaky." If that is impractical, further emphasize that your results are bounding extremes rather than describing the full continuum of immune states.

• **Predictive Validation:** Clarify in the text whether the intent is purely conceptual vs. predictive. If you aim to show any predictive power, consider including a short out-of-sample forecast (without re-fitting) to demonstrate that the immunity assumption influences longer-term results.

• **Age/Risk Stratification:** If there is any quick and easy way to include at least a two-group approach (e.g., "low-risk" vs. "high-risk"), that would strengthen your case. If not, I encourage a more direct discussion in the text explaining why ignoring heterogeneity does not severely bias the main insights.

• **Model-Selection Criterion:** A formal comparison (e.g., "leaky vs. polarised likelihood, DIC, etc.") is not strictly mandatory, but it would be valuable. At the very least, stating explicitly that both models can fit the observed data well—and thus are not distinguishable on short-term hospital admissions alone—helps contextualize your conclusion.

To summarise the changes made, we accounted for all points, excepted for the risk stratification, for which we further stress the associated limitation in the main text.

Overall, I appreciate the improvements: the revised paper is clearer about assumptions, shows more sensitivity testing, and acknowledges that real-world immunity can be far more nuanced. With a bit more emphasis on how these two formalisms bracket the true scenario—and possibly a brief numerical or comparative extension—this paper would strongly illustrate why immunological assumptions matter for long-term epidemic projections.
I look forward to seeing the next iteration.

Thank you for the detailed feedback that improved the overall quality of the manuscript. We appreciate it.

Reviewer #3

It is a pleasure to see that the authors have restructured the exposition of the paper, and that it adds a lot of value in terms of clarity and completeness. It is also commendable that the authors went into detail when answering all the points raised by the reviewers. In my opinion, all comments have been sufficiently addressed, especially the exposition of the methods – in the Appendix, but also the main part –, the sensitivity analysis, and the comparison between the leaky and polarised formalism.

I have a few remaining minor points:

In line 101, you write "Assuming $\tau = 0$, we have ...". But what you assume is that $q_l(0) = q_p(0) = 1$ at $\tau = 0$. It should be phrased accordingly, i.e. "We assume $q_l(0) = q_p(0) = 1$," as otherwise your assumption would refer to τ.

Thank you, we corrected it.

You write "The number of remaining uninfected individuals ...", but it seems as if q_l and q_p are in fact proportions of uninfected individuals? (if $q_l(0) = q_p(0) = 1$)

Thank you, we corrected it.

On the same note, only if q_l and q_p represent proportions do your calculations imply that $q_l(\tau) <= q_p(\tau)$, as $q_l(\tau) <= 1$ (otherwise your induction step from 1 to 2 doesn't work). You should add a comment on this.

You are right in that this is a fundamental assumption.

We added a sentence in the Supplementary information stating that the induction from step 1 to step 2 was possible because we work with proportions, which implies that $q_\ell \leq 1$.

In the Results section, in Equations (1) and (2), q_l and q_p at time $\tau + 1$depend on the values of q_l, q_p respectively, and both also on ξ, at time τ. Yet, in the Appendix in (S1), (S2) etc. you falsely write ξ evaluated at the time step $\tau + 1$.

Thank you for noticing this. Yes, you are absolutely correct and ξ was supposed to be evaluated at time τ. This does not affect the conclusions drawn in the main text.

In the Appendix on the first page, you have some Equations not yet linked, showing up as ??.

We corrected the error. Thanks for pointing this out.

With these comments addressed, I can fully recommend this article for publication.

We thank you again for your positive impact on this manuscript.
