# Supplementary material for: Leaky or polarised immunity: Non-Markovian modelling highlights the impact of immune memory assumptions
Source: PLoS Comput Biol. 2025 Aug 19;21(8):e1013399. doi: 10.1371/journal.pcbi.1013399 (PMC12407549; doi:10.1371/journal.pcbi.1013399)
Supplement: Attachment — Submitted filename: PCOMPBIOL-D-25-00077_R2(JUN30).pdf [file pcbi.1013399.s008.pdf]

Referee report on PCOMPBIOL-D-25-00077_R2

We thank the authors for providing a revised version of their manuscript. The work addresses an important issue – how different assumptions about immune memory affect epidemic projections. While the revision clarifies several aspects, a number of technical and conceptual issues remain, as detailed below.

Some mathematical assumptions are not fully stated, several model equations need clarification, and certain empirical claims are either overstated or insufficiently justified. Addressing the points below will improve the manuscript's rigour and transparency.

Major comments

M1. Implicit monotonicity assumption in Sect. 2 proof. The induction that establishes shorter reinfection intervals under leaky immunity relies on $\xi(\tau)$ being non-increasing in τ; this assumption should be stated explicitly and discussed.

M2. Force-of-infection formula (Eq. (5)). The proposed logistic form has no derivation or citation. The standard renewal equation would use $\Lambda_t = R\,c_t \sum_i \omega_i (I + J)_{t-i,i}$. Please justify the chosen expression or revert to the canonical form.

M3. Double use of the hospitalisation probability p_h (Eqs. (3)–(4)). Compartment J is defined as "always hospitalised"; multiplying the inflow by p_h apparently applies the filter twice, leading to an undercount of admissions. Clarify whether J already represents severe cases *after* hospital admission.

M4. Assumption of full prior immunisation (Methods). The model sets the entire French population as immunised on 26 Nov 2021. Vaccination coverage at that date was clearly $<100\,\%$. Please justify or relax this assumption and discuss its impact on the results.

M5. Omission of mortality. Ignoring COVID-19 deaths removes a depletion channel for susceptibles and may inflate reinfection counts. Briefly quantify the expected bias or incorporate a death flow.

M6. Terminology: "SIRI" vs. SIRS. The path $S \to I \to R \to I$ with immediate loss of immunity is normally labelled SIRS with $\sigma = 1$. Consider adopting the standard name or motivate the new acronym.

M7. Undefined or inconsistently named symbols. Variables N, c_t, R_c and several subscripts (e.g. t, i) appear without definition. A table of symbols would help ensure reproducibility.

Minor comments and technical corrections

m1. Inconsistent terms for the same concept: "all-or-nothing", "polarised" and "polarized" are all used; please choose one and standardise.

m2. References [42] and [43] appear to cite the same article; merge or clarify.

Recommendation

With the above issues resolved—particularly the explicit statement of assumptions, clarification of equations, and correction of empirical claims—the manuscript would make a valuable contribution. I look forward to reviewing a thoroughly revised version.
