# Supplementary material for: Leaky or polarised immunity: Non-Markovian modelling highlights the impact of immune memory assumptions
Source: PLoS Comput Biol. 2025 Aug 19;21(8):e1013399. doi: 10.1371/journal.pcbi.1013399 (PMC12407549; doi:10.1371/journal.pcbi.1013399)
Supplement: Attachment — Submitted filename: RESPONSE_R3.pdf [file pcbi.1013399.s009.pdf]

Response to reviewers

You will find our answers (in black) to the reviewer's remarks (in blue).

Reviewer #2

We thank the authors for providing a revised version of their manuscript. The work addresses an important issue – how different assumptions about immune memory affect epidemic projections. While the revision clarifies several aspects, a number of technical and conceptual issues remain, as detailed below.
Some mathematical assumptions are not fully stated, several model equations need clarification, and certain empirical claims are either overstated or insufficiently justified. Addressing the points below will improve the manuscript's rigour and transparency.

Major comments

M1. Implicit monotonicity assumption in Sect. 2 proof. The induction that establishes shorter reinfection intervals under leaky immunity relies on $\xi(\tau)$ being non-increasing in τ; this assumption should be stated explicitly and discussed.

We are not sure we fully understand this comment sionce this assumption was not implicit, as in the previous version we state multiple times that the function $\xi(\tau)$ is "decreasing".

Nevertheless, to avoid any misunderstanding, we extended the sentence in line 106 to make it clearer: "*This proves individuals are reinfected more quickly through leaky than polarised immunity—assuming the function $\xi(\cdot)$ is decreasing.*"

M2. Force-of-infection formula (Eq. (5)). The proposed logistic form has no derivation or citation. The standard renewal equation would use $\Lambda_t = Rc_t \sum_i \omega_i (I+J)_{t-i,i}$. Please justify the chosen expression or revert to the canonical form.

We now cite one of our previous works, where this particular derivation is justified and detailed.

M3. Double use of the hospitalisation probability p_h (Eqs. (3)–(4)). Compartment J is defined as "always hospitalised"; multiplying the inflow by p_h apparently applies the filter twice, leading to an undercount of admissions. Clarify whether J already represents severe cases after hospital admission.

We are not sure that we understand this comment since the quote "J is defined as 'always hospitalised'" is not from our manuscript. Instead, we wrote that J represent "severe infections that always lead to hospitalisation". Furthermore, our Figure 3 displays the model flowchart, which should avoid confusions regarding the model structure.

Still related to Reviewer #2's concern, equations (3) and (4) do not involve compartment J. Nevertheless, in case the reviewer mistook index j for variable J, we decided to replace the index j by the index k to avoid any confusion.

M4. Assumption of full prior immunisation (Methods). The model sets the entire French population as immunised on 26 Nov 2021. Vaccination coverage at that date was clearly <100%. Please justify or relax this assumption and discuss its impact on the results.

This point seems to disregard the previous round of reviews as this issue was already raised in Round 1 by Reviewers #1 and #2, and addressed in our response. In particular, we already included a sensitivity analysis (Appendix G) that pleased both Reviewers.

M5. Omission of mortality. Ignoring COVID-19 deaths removes a depletion channel for susceptibles and may inflate reinfection counts. Briefly quantify the expected bias or incorporate a death flow.

Again, this point was already raised (and as a "Minor point") in the first round of review by Reviewer #2. We addressed and acknowledged this limitation, which was not an issue in Round 2. In particular, we write in the Discussion that "*We also neglect death in our model, which is not linked to the choice of the immunity formalism, although it could have marginally diminished the number of reinfections.*"

M6. Terminology: "SIRI" vs. SIRS. The path $S \to I \to R \to I$ with immediate loss of immunity is normally labelled $SIRS$ with $\sigma = 1$. Consider adopting the standard name or motivate the new acronym.

We respectfully disagree with the reviewer and are, again, surprised to see this point raised for the first time during revision Round 3. Indeed, this is precisely our motivation as we study the differences between polarised models ($SIRS$-like) compared to the leaky ones ($SIRI$-like). This is stated in the Introduction:

> *For the polarised assumption, the SIRS model is the simplest formulation under this assumption. For the leaky assumption, we postulate that a recovered individual can be reinfected directly without going back into the susceptible compartment S [36]. The path followed by an individual is then $S \rightarrow I \rightarrow R \rightarrow I$, so we refer to this model as SIRI (Figure 1C).*

In addition to this, an appropriate model with an "immediate loss of immunity" would be an SIS model, and not a "$SIRS$ with $\sigma = 1$".

> **M7. Undefined or inconsistently named symbols.** Variables N , c_t, R_c and several sub- scripts (e.g. t, i) appear without definition. A table of symbols would help ensure reproducibility.

We are surprised, as all the terms mentioned are defined in the sentences that follow their introduction, except for the term R_c... which is not used in our manuscript. The subscripts are also defined for each variable as soon as they are introduced.

> **Minor comments and technical corrections**

> **m1.** Inconsistent terms for the same concept: "all-or-nothing", "polarised" and "polarized" are all used; please choose one and standardise.

We double checked and did not use the term "polarized" in the revised version (it wass always spelt "polarised"). Furthermore, the only time we use the term "all-or-nothing" is when we write "*We refer to this all-or-nothing pattern as polarised, following a terminology introduced by [33].*" Therefore, we were not able to find the inconsistencies referred to by the reviewer.

> **m2.** References [42] and [43] appear to cite the same article; merge or clarify.

We corrected it, thank you.

Recommendation

With the above issues resolved—particularly the explicit statement of assumptions, clarification of equations, and correction of empirical claims—the manuscript would make a valuable contribution. I look forward to reviewing a thoroughly revised version.

We hope that our manuscript is now acceptable for publication.
