# Supplementary material for: Leaky or polarised immunity: Non-Markovian modelling highlights the impact of immune memory assumptions
Source: PLoS Comput Biol. 2025 Aug 19;21(8):e1013399. doi: 10.1371/journal.pcbi.1013399 (PMC12407549; doi:10.1371/journal.pcbi.1013399)
Supplement: Attachment — Submitted filename: PCOMPBIOL-D-25-00077(Review Report).docx [file pcbi.1013399.s002.docx]

**PCOMPBIOL-D-25-00077**

Title: Leaky or polarised immunity: non-Markovian modelling highlights the impact of immune memory assumptions

**Review Report**

I would like to begin by thanking the authors for their thorough and thought-provoking work on “Leaky or polarised immunity: non-Markovian modelling highlights the impact of immune memory assumptions.”

I appreciate the time and effort that went into developing the non-Markovian model, performing the numerical experiments, and analyzing different immunity assumptions. In my opinion, this study addresses an important question related to COVID-19 modeling: whether immunity is better captured by a “leaky” or a “polarized” mechanism. Below, I offer my comments and suggestions, which I hope will be useful as you revise the manuscript.

1. Major Points

1.1 Single population group (no age or comorbidity stratification)

In my opinion, one of the key limitations of the current model is the absence of any population stratification by age or risk factors. Given that COVID-19 severity, hospitalization rates, and reinfection risks vary significantly with age and comorbidities, modeling the entire population as one homogeneous group may bias the results.

- The average risk of hospitalization or severe outcomes could be quite different from reality if a large proportion of hospitalized individuals actually belong to higher-risk subgroups. In my opinion, this limitation could affect the interpretation of the results regarding reinfection rates and hospitalization patterns.

- I believe it would strengthen the paper to include at least two or three age/risk strata. If doing so is not feasible in the current version, you might consider discussing thoroughly how homogeneous assumptions could skew your results.

1.2 Assumption of “fully immunized population” before Omicron

In the section “Modelling the initial waves of the Omicron variant,” the text suggests that everyone was already immunized (by either Delta infection or vaccination) prior to Omicron’s emergence. In my opinion, this may be overly simplistic because, in reality, there was still a fraction of people without prior vaccination or infection, and vaccines were not 100% effective against Delta.

- This assumption could distort the susceptible and reinfection dynamics during the Omicron wave, leading to a mismatch with real-world situations.

-	I think it would be important to justify this assumption more comprehensively or conduct a sensitivity analysis. Discussing the robustness of your conclusions under different levels of pre-Omicron immunity would also be helpful.

1.3 Strict comparison between “leaky” and “polarized,” with no hybrid models

In my view, comparing two extreme forms of immunity—leaky (partial protection for all) vs. polarized (some are fully protected, others are not at all)—is certainly insightful. However, there is empirical evidence (e.g., for COVID-19) that real populations might exhibit hybrid responses, with gradations between these extremes.

- By focusing only on the two polar extremes, the numerical results might overstate the differences. In my opinion, many real-world scenarios likely fall in between.

- I suggest, at minimum, a deeper discussion of possible hybrid scenarios and the biological rationale for focusing on these two extremes. Highlight that leaky and polarized are presented as limit cases—if you do not model hybrid immunities, I believe it is necessary to justify clearly why these extremes suffice for the study.

1.4 A posteriori calibration of contact rate without predictive comparison

The manuscript mentions a Bayesian fitting procedure in which the contact rate is adjusted every ~20 days to match hospitalization data. In my opinion, this approach may obscure structural uncertainties in the model: with sufficient flexibility in the contact rate, almost any epidemic curve might be replicated, making it hard to distinguish the impact of leaky vs. polarized immunity.

- This could diminish the predictive power of the model, especially if you cannot show how well the model performs in periods not used in the calibration.

- I would recommend including some predictive checks or out-of-sample validation to ensure that the model’s ability to match hospitalization curves is not merely a byproduct of repeatedly adjusting the contact rate. A stronger discussion of how these calibration intervals might affect the main conclusions would also be valuable.

1.5 Lack of direct comparison with empirical data

- The article references certain observational studies (e.g., Lind et al. [2023]) that suggest leaky immunity in specific outbreak settings. In my opinion, however, the manuscript does not explicitly confront the model’s outputs with real-world data or attempt to empirically differentiate which immunity regime is more plausible.

- If the goal is to elucidate leaky vs. polarized immunity in practice, I believe some additional validation or at least a discussion of possible empirical markers for each regime could greatly enhance the paper’s relevance.

- In my view, I encourage you to propose ways to validate leaky vs. polarized immunity assumptions beyond the single observational reference. It would help readers connect the model results with real data more concretely.

2. Minor Points

2.1 Potential confusion around terminology

- Example

The phrase “basic reproduction number (R0) of Omicron” is used in the modeling section. Strictly speaking, R0 is defined in a fully susceptible population, so calling it an R0 for Omicron can be misconstrued if the population is not fully susceptible.

In my opinion, it would be clearer to label this parameter an “effective reproduction rate” or clarify that it is a hypothetical scaling factor.

2.2 Functions for waning and immune escape

- The manuscript refers to multiple functional forms for waning immunity and immune escape in appendices, but the main text does not go into much detail.

I think providing more biological or empirical justification for each function in the main text would improve clarity. Even just a short paragraph summarizing the rationale (linearity, sigmoid decay, etc.) would help.

2.3 No differentiation between vaccine-derived and infection-derived immunity

-	The model lumps all “immunized” individuals together, without distinguishing vaccine-induced vs. infection-induced immunity. In reality, these might have different waning kinetics and protective profiles.

In my opinion, the manuscript should acknowledge and briefly discuss this limitation. If further stratification is impractical, explaining why it is not crucial for your primary conclusions would be beneficial.

2.4 Absence of mortality in the model

The model flow is infection → hospitalization → recovery, with no explicit compartment or rate for COVID-19 fatalities.

Even though COVID-19 mortality may be relatively small compared to total infections, in my opinion, it could influence long-term dynamics. If mortality is omitted, a brief statement about its potential impact would be appropriate.

3. Overall Recommendation: Major Revisions

When I weigh these points, I conclude that there are enough substantive issues—particularly around population stratification, the assumption of universal pre-Omicron immunity, the dichotomy between leaky vs. polarized, and the a posteriori calibration of contact rates—that, in my opinion, this manuscript would benefit from major revisions.

-	I want to emphasize that I genuinely appreciate the sophisticated non-Markovian framework, the focus on exploring different immunity mechanisms, and the numerical experiments presented. I believe the paper addresses a timely question and has strong potential.

-	In my opinion, the main text should discuss more thoroughly the key assumptions (e.g., homogeneous population, no mortality, universal immunization before Omicron) and their potential impact on the interpretation of the results. If it is not feasible to change the model structure now, a robust justification and discussion of limitations would still greatly strengthen the manuscript.

4. Suggestions to Improve Clarity and Organization

1. I realize that in detailed modeling papers, it is common to place extensive derivations and supplementary analyses in the appendices. However, in my opinion, some critical justifications (for instance, the choice of waning functions, contact-rate calibration details) should be outlined more explicitly in the main text so that the reader does not need to constantly switch to the supplementary material.
2. I notice that some figure legends are quite long, almost repeating sections of the discussion or methods. In my opinion, figure legends should provide all essential information to interpret each figure but should not become overly redundant. Perhaps you could move lengthy methodological explanations to the main text or the appendix, keeping figure legends more concise but still self-contained.
3. In my perspective, the manuscript might flow more smoothly if references to modeling assumptions and their justifications were systematically included in the main “Methods” and “Results” sections, with clear pointers to the appendices for further mathematical details.

- For instance, “We use a flexible Bayesian approach to recalibrate the contact rate every 20 days (see Appendix X for details).”

- This approach would keep the main text succinct but still let readers see the rationale without flipping back and forth too often.

Overall, I believe that incorporating these adjustments and clarifications—especially regarding stratification, justification of assumptions, discussion of hybrid immunity, and more robust calibration checks—will significantly strengthen the manuscript. In my opinion, once these major points are addressed, the paper will be in a much better position to communicate its valuable findings to the readership of PLOS Computational Biology.

I thank the authors again for their work and for the opportunity to review this submission. I hope my comments will be taken in the constructive spirit intended, and I look forward to seeing a revised version of this interesting study.
