# Supplementary material for: Leaky or polarised immunity: Non-Markovian modelling highlights the impact of immune memory assumptions
Source: PLoS Comput Biol. 2025 Aug 19;21(8):e1013399. doi: 10.1371/journal.pcbi.1013399 (PMC12407549; doi:10.1371/journal.pcbi.1013399)
Supplement: Attachment — Submitted filename: PCOMPBIOL-D-25-00077(Review Report) (2).docx [file pcbi.1013399.s005.docx]

**PCOMPBIOL-D-25-00077**

**Title**: *Leaky or polarised immunity: non-Markovian modelling highlights the impact of immune memory assumptions*

**Review Report (Second Round)**

I would like to thank the authors again for their thoughtful revisions and clarifications to the manuscript. In my earlier review, I raised several major points, which I will revisit here in light of the new version. Overall, the manuscript has improved in terms of clarity and explicit acknowledgment of potential limitations. However, a few substantive issues remain and merit the authors’ consideration before final acceptance.

**1. Single Population Group (No Age or Comorbidity Stratification)**

**Original Concern** (1.1):
I noted that modeling the entire population as homogeneous may bias results, since COVID-19 severity, hospitalization risks, and reinfection dynamics vary substantially by age and comorbidities.

**Authors’ Response and Revisions**

- The authors now discuss in the text that they assume a single age group and justify that “the attack rate was similar across age groups in France.”
- They argue this choice keeps the model simpler, emphasizing the difference between “leaky” vs. “polarized” immunity rather than the heterogeneities of subpopulations.

**What Remains**

- While the authors do acknowledge this limitation, the paper still provides limited quantification or sensitivity analysis on how much ignoring age/risk structure might skew hospitalization estimates.
- In practice, hospital admissions skew older, so the time to reinfection and probability of severe disease could differ in a multi-strata model. In my opinion, a short statement or additional sensitivity test (even if minimal) could strengthen the argument that “the homogeneous approach does not invalidate the key result.”

**2. Assumption of “Fully Immunized Population” Before Omicron**

**Original Concern** (1.2):
I questioned whether assuming that *everyone* was immunized by Delta or vaccination before Omicron might distort subsequent dynamics, since a fraction remained non-immune in reality.

**Authors’ Response and Revisions**

- The revised manuscript explicitly includes a sensitivity analysis (see their Appendix G) where the initial “recovered” compartment is populated differently (uniform or modes at different times).
- They note that this helps illustrate that the exact fraction of pre-Omicron immunity does not drastically alter the qualitative difference between leaky and polarized assumptions.

**What Remains**

- While the new text clarifies the setup, it might still be worth more explicitly acknowledging that a small fraction truly had zero prior protection.
- Overall, the authors’ sensitivity checks appear sufficient to show that the key result—shorter time to reinfection under “leaky” vs. “polarized”—is robust under multiple initial distributions.

**3. Strict Comparison Between “Leaky” and “Polarized,” With No Hybrid Models**

**Original Concern** (1.3):
Real-world immunity likely lies between these two extreme assumptions. I suggested either (a) a numerical exploration of intermediate/hybrid immunity or (b) a deeper discussion justifying the focus on these two extremes.

**Authors’ Response and Revisions**

- The authors add some discussion referencing works on partial or “immune boosting” frameworks (e.g., Park et al. [2023]) and note that these extremes capture two endpoints of imperfect immunity.

**What Remains**

- Despite the discussion, there is still no direct simulation or parametric test of a “mixed” approach (e.g., 50% fully protected, 50% partially protected).
- I recognize that adding this would expand the study’s scope considerably, but **even a short numerical experiment** or scenario analysis for a “fraction leaky vs. fraction polarized” would be valuable to show whether real data might fall between the extremes.

**4. A Posteriori Calibration of Contact Rate Without Predictive Comparison**

**Original Concern** (1.4):
By re-fitting the contact rate in 20-day windows, the model can accommodate many dynamics. I asked for out-of-sample checks or predictive validation to ensure that repeated calibration does not mask fundamental differences between immunity assumptions.

**Authors’ Response and Revisions**

- In the supplement, they analyze the sensitivity of results to 10-day vs. 20-day fitting windows, and briefly compare the fitted transmission rate to Google Mobility Data.
- They argue this partly addresses the risk of overfitting because they see some consistency with exogenous mobility trends.

**What Remains**

- While these additions help, we do not see a strict out-of-sample forecast (fitting up to date T, then predicting T+1 onward). Consequently, the difference in predictive power between leaky vs. polarized remains somewhat unclear.
- If the manuscript’s main objective is more conceptual than predictive, this may be acceptable. Nonetheless, clarifying that further predictive validations were not the focus might help.

**5. Lack of Direct Comparison With Empirical Data**

**Original Concern** (1.5):
I suggested that the authors try to differentiate which immunity regime fits the data better, perhaps via a likelihood-based comparison or referencing more than one observational study.

**Authors’ Response and Revisions**

- They do point to Lind et al. [2023] and note that certain data suggest real SARS-CoV-2 immunity is “leakier” than previously thought.
- The new version also more openly discusses how real data might help identify leaky vs. polarized assumptions in practice.

**What Remains**

- The paper still does not present, for example, an AIC or WAIC comparison between the two models applied to the same dataset. We only see that **both** formalisms can reproduce hospitalization curves once contact rates are adjusted.
- A formal model-selection approach might still be out of scope, but at least acknowledging that the two frameworks might be empirically discriminated in longer time series or in controlled cohorts is helpful.

**Summary of Recommendations**

- **Hybrid Immunity**: If feasible, add a brief scenario or appendix section illustrating what happens when a fraction of individuals has “polarized” immunity and another fraction has “leaky.” If that is impractical, further emphasize that your results are bounding extremes rather than describing the full continuum of immune states.
- **Predictive Validation**: Clarify in the text whether the intent is purely conceptual vs. predictive. If you aim to show any predictive power, consider including a short out-of-sample forecast (without re-fitting) to demonstrate that the immunity assumption influences longer-term results.
- **Age/Risk Stratification**: If there is any quick and easy way to include at least a two-group approach (e.g., “low-risk” vs. “high-risk”), that would strengthen your case. If not, I encourage a more direct discussion in the text explaining why ignoring heterogeneity does not severely bias the main insights.
- **Model-Selection Criterion**: A formal comparison (e.g., “leaky vs. polarised likelihood, DIC, etc.”) is not strictly mandatory, but it would be valuable. At the very least, stating explicitly that both models can fit the observed data well—and thus are not distinguishable on short-term hospital admissions alone—helps contextualize your conclusion.

Overall, I appreciate the improvements: the revised paper is clearer about assumptions, shows more sensitivity testing, and acknowledges that real-world immunity can be far more nuanced. With a bit more emphasis on how these two formalisms bracket the true scenario—and possibly a brief numerical or comparative extension—this paper would strongly illustrate why immunological assumptions matter for long-term epidemic projections.

I look forward to seeing the next iteration.
